# Genome Profiling (GP) Method Based Classification of Insects: Congruence with That of Classical Phenotype-Based One

**DOI:** 10.1371/journal.pone.0023963

**Published:** 2011-08-31

**Authors:** Shamim Ahmed, Manabu Komori, Sachika Tsuji-Ueno, Miho Suzuki, Akinori Kosaku, Kiyoshi Miyamoto, Koichi Nishigaki

**Affiliations:** 1 Graduate School of Science and Engineering, Saitama University, Saitama, Japan; 2 Laboratory of Clinical Sciences, Dokkyo Medical University, Tochigi, Japan; 3 Laboratory of Medical Sciences, Dokkyo Medical University, Tochigi, Japan; King Abdullah University of Science and Technology, Saudi Arabia

## Abstract

**Background:**

Ribosomal RNAs have been widely used for identification and classification of species, and have produced data giving new insights into phylogenetic relationships. Recently, multilocus genotyping and even whole genome sequencing-based technologies have been adopted in ambitious comparative biology studies. However, such technologies are still far from routine-use in species classification studies due to their high costs in terms of labor, equipment and consumables.

**Methodology/Principal Findings:**

Here, we describe a simple and powerful approach for species classification called genome profiling (GP). The GP method composed of random PCR, temperature gradient gel electrophoresis (TGGE) and computer-aided gel image processing is highly informative and less laborious. For demonstration, we classified 26 species of insects using GP and 18S rDNA-sequencing approaches. The GP method was found to give a better correspondence to the classical phenotype-based approach than did 18S rDNA sequencing employing a congruence value. To our surprise, use of a single probe in GP was sufficient to identify the relationships between the insect species, making this approach more straightforward.

**Conclusion/Significance:**

The data gathered here, together with those of previous studies show that GP is a simple and powerful method that can be applied for actually universally identifying and classifying species. The current success supported our previous proposal that GP-based web database can be constructible and effective for the global identification/classification of species.

## Introduction

Identification and classification of species are fundamental to biology and biotechnology; traditionally, these processes were carried out for each biological domain by trained experts who used phenotype-based methods. Even with the advent of genome-sequencing methodologies, this remains basically true. As a consequence, advances in classification-based fields have been delayed due to dependence on the relatively small number of experts capable of performing these laborious and time-consuming phenotypic analyses. Many attempts have been made to develop methods that reduce these difficulties. For example, internet-assisted database systems and automatic data-processing have been developed for use in identifying and annotating species-specific phenotypic or genotypic traits [1], [2]. Inevitably, organisms only with a wealth of morphological features that can be used for classification, for example insects, vertebrates, and plants, have well-established systems for the identification of species using publicly accessible databases, although these are still not fully systematized and are now under discussion [3], [4]. In this study, we analyzed insect species as a representative group of organisms as these have been extensively and energetically studied from the morphological standpoint; this has enabled us to compare classification results between the phenotype- and the genotype (genome)-based approaches, which had been wanted. Indeed, the need for such methods has increased due to the upsurge in worldwide transportation of goods and people, which has raised fears of worldwide pandemics caused by known or unknown microorganisms. Unfortunately, there is at present no validated system for identifying and classifying species that is universally applicable. Over the past 30 years, however, DNA sequence data have been accumulated from an increasingly wide range of organisms and have been exploited for species identification and classification [5]–[7]. The sequencing approach based on 16S/18S rDNA is one of the most commonly used and widely accepted [8], [9]. However, the information produced is well-known to be often insufficient to provide a unique identification/classification of a species or to explain phylogenetic relationship of species [9], [10]. This limitation has stimulated development of supplementary approaches such as multi-loci sequence typing (MLST) [11], or use of the whole genome sequence to identify the organism. Although the latter approach has become more realistic because of the development of “next-generation” sequencers, which can perform gigabase sequencing per day, it is unlikely that this will become the standard approach for identifying and classifying species, much like a jet plane cannot replace the function of a bicycle.

**Figure 1:**
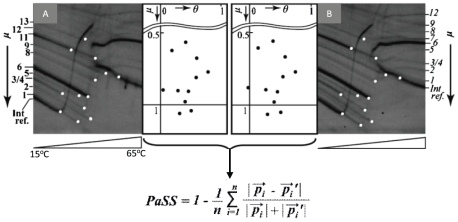
Genome profiles and spiddos patterns. DNA fragments obtained by random PCR are layered at the top of a slab gel; the fragments migrate downward with a characteristic curvature caused by the temperature gradient. Feature point(s) for each DNA fragment, i.e., the initial melting point from double-stranded to single stranded one, are indicated by the white dot(s) in panels A and B for genomes A and B, respectively. Species identification dots (spiddos), shown in the panels adjacent to A and B, are obtained by normalizing the coordinates of the feature points with those of an internal reference DNA fragment. Spiddos thus obtained are genome-specific and can be used to calculate the pattern similarity score (*PaSS*) or genomic distance (i.e., 1−*PaSS*) to construct a phylogenetic tree. Gel images are taken from *Chemistry Letters* [14] and modified with permission.

An alternative approach termed genome profiling (GP: see Fig. S1) was developed in an attempt to circumvent the limitations of the sequencing methods, and was initially shown to be able to discriminate between species [12] and was subsequently validated in a range of organisms [13]–[18]. GP consist of i) the random PCR step which enables to obtain DNA fragments from the whole genome in a random sampling mode, and ii) temperature gradient gel electrophoresis (TGGE), used for separating obtained DNA fragments. The power of analysis comes from the fact that TGGE utilizes both mobility (size information) and temperature-induced structural transition of DNA fragments (sequence-dependent information) [19], [20] and thus, making the approach highly resolvable and powerful one. As GP employs a sophisticated measure to eliminate experimental variables (computer-aided normalization with internal references), it is consistently reproducible (Fig. 1). GP quantifies the differences between the genomes of different species, and has also been employed to measure the degree of genomic DNA damage resulting from exposure to UV or chemical mutagens [14], [15]. Overall, GP measures genomic distances. It has been used successfully to identify a wide spectrum of species, from viruses to vertebrates [16], and has also been shown to be of value for the classification of species [17], [18]. In this study, we further tested these capabilities by applying GP to a large number of insect species and comparing the results with those obtained by the 18S rDNA sequencing approach for the same set of insects. This is the first case for the GP method to be analytically compared with the genotyping approach (18S rDNA) by employing morphologically well-studied organisms (insects of 26 species).

## Results and Discussion

**Figure 2:**
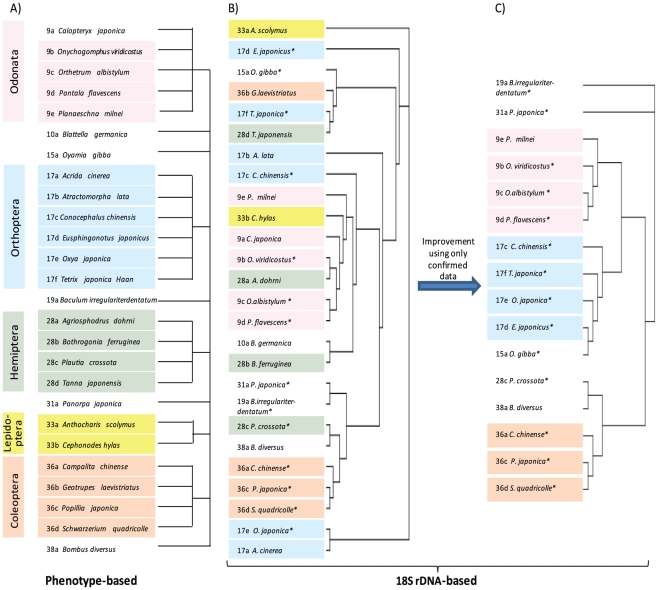
Sequence-based phylogenetic trees of insects compared to the phenotype-based tree. The phenotype-based tree (A) was drawn using the data presented by Iwatsuki *et al.*, (1960), which appeared in the Biological Encyclopedia (published by Iwanami, Tokyo, Japan, 1900). The numbering put at each front came from the taxon number of probable evolutionary order of appearance. Species that belong to the same Order are shown in the same color. (B) The tree obtained using insect 18S rDNA sequences is depicted similarly as in panel A. (C) The insect 18S rDNA sequences which were confirmed against the NCBI database, i.e., those sequences which appear in the NCBI database with the congruence of more than 97%, were used to draw this tree.

In this study, 26 species of insects belonging to the orders Odonata (dragonfly), Orthoptera (grasshopper), Hemiptera (cicada), Lepidoptera (butterfly), Coleoptera (beetle), or to related taxa were selected for analysis. The 18SrDNA (∼550 nucleotides) of each species was first sequenced and a phylogenetic tree was constructed using ClustalW (Fig. 2). Although the sequencing was performed with particular care (corroborated by double sequencing) and all of the sequences obtained were confirmed to be 18S rDNA, the phylogenetic tree showed poor correspondence (Congruence value, *V_c_* = 0.06 and *V_c_′* = 0.19; where the congruence values *V_c_* and *V_c_*′ are a kind of measure to evaluate the similarity between two (phylogenetic) trees, introduced in relation to this study (see Text S1 as appendix paper for detail). *V_c_* is the direct measuring while *V_c_*′ is obtained after a coarse-graining process about complicated trees.) with the conventional tree based on phenotypic characters (Fig. 2, Panels A and B). To eliminate the possibility that the poor correspondence resulted from contamination by non-insect 18S rDNA sequences, we performed a BLAST homology search for the obtained sequences in the NCBI database. We eliminated any sequences that showed a sequence identity of less than 97% with the database. This had the effect of selecting only those sequences that have been confirmed in two independent sequencing analyses (here and NCBI). When the tree was reconstructed using the 16 species thus selected, the correspondence between the 18S rDNA and classical trees was very much improved up to the *V_c_* of 0.26 and *V_c_′* of 0.73 (Fig. 2, Panel C). This comparison indicates that the DNA sequence quality needs to be sufficiently high to obtain reliable phylogenetic trees and, second, that tree-making based on the high quality 18S rDNA sequences can provide a result that is basically consistent with that obtained by the classical phenotype-based approach though not complete congruence. The latter conclusion is somewhat unexpected since consistency between classical and 18S rDNA sequence-based phylogenetic trees is believed to be moderate at best unless artificial selection or statistical operations are performed to make them congruent, as discussed later [9], [10].

**Figure 3:**
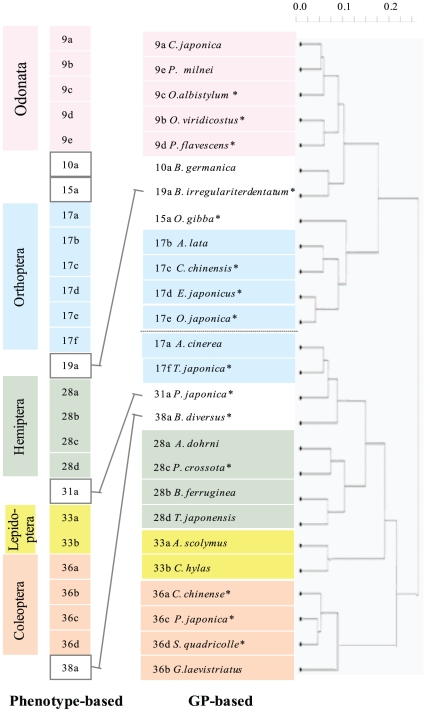
GP-based phylogenetic tree. The members of the various Orders formed monophyletic clusters and showed good correspondence to the phenotype-based tree (i.e., the classical tree). Species that belong to the same Order are shown in the same color. Non-correspondences between the classical tree and the GP-based tree are indicated by lines which show the possible realignments necessary to make the two classifications match completely. The superscript star symbol indicates species that are present in Fig 2, panel C.

The 26 insect species were then subjected to GP analysis and the data used to construct a phylogenetic tree (Fig. 3). The phylogenetic tree produced by GP was consistent with the classical tree and showed remarkably better congruence (*V_c_* = 0.24 and *V_c_′* = 0.71) than was obtained using the 18S rDNA sequencing data for all 26 species (Fig. 2, Panel B). Especially this is the case with the *V_c_′* values (0.19 for 18S rDNA sequencing vs 0.71 for the GP approach). This congruence was unexpected as the GP experiments were performed using a single probe (thus, being labor-saving). Similar results have already been reported for groups with a smaller number of members such as 12 species of plants, 14 species of insects, and 14 species of fish [17] (Fig. S2 and Table S1). In combination, these data indicate that GP can classify species simply and robustly, and conserve congruence with phylogenetic trees constructed by the classical (phenotype-based) approach. The reason for the success of the GP approach is likely due to the fact that trees generated by a set of GP data are less sensitive to experimental errors as experimentally shown [21]. This indeed seems to be the case for the results obtained here since it was not necessary to perform a confirmation process as was required for the 18S rDNA sequencing-based results (Fig. 2C).

It is inevitable that the analyses will be subject to experimental errors to a greater or less extent. Therefore, the robust nature of the GP method in providing a correct phylogenetic tree despite such experimental variables is a considerable advantage and indicates that the method must be very powerful as a universal classification method. In theory, it should be possible to increase the reliability of the analysis by performing an increased number of experiments [13]. A particular advantage of the GP approach is that it is less costly and less laborious (Table S2 and Table S3) than the 18S rDNA approach since it comprises only PCR, gel electrophoresis, and image processing steps (Fig. S1). Although almost all of the species assigned to the order Orthoptera were positioned together, species 17a and 17f formed a separate cluster (painted blue in Fig. 3). This result is rather temporal due to possible errors inevitably contained in the GP method. Nevertheless, this nature of Orthoptera order, i.e., being less collective, may reflect some disorder in their genomes, serving as a working hypothesis.

The GP method also has the merit that it can generate data (spiddos) that can be obtained from any organism and can be processed easily to measure the genomic distance between two species (Fig. 1). This fact was confirmed by applying the GP method to the classification of insects here and will eventually allow us to construct a database that is universally applicable; a preliminary attempt to achieve this goal has already been initiated [22]. Since the spiddos can be directly obtained from a gel image with the help of an internet database service as shown in Figure 1, any scientist can easily obtain a set of spiddos for a species of interest, and these can be registered and used for identification and classification. Therefore, the spiddos can be extracted from gel images using only an internet service such as On-web GP [22], [23]. By employing the spiddos as a form of species index, we can collect and integrate all of the properties associated with a particular organism without knowing its identity (i.e., without an expert's painstaking identification process) [22]. In other words, we have acquired another reliable label for each organism that can be obtained without relying on experts in classification. Obviously, this approach has the potential to be a great influence across many biological fields where species identification is important. In particular, GP must be most beneficial to microbiology related studies since confirmation of species identity can be almost equivalent to an independent, painstaking research. Reassuringly, successful applications of GP to microbial organisms have been reported [17], [18], [24], [25].

From the entomological viewpoint, our comparison of the phylogenetic trees produced by the classical phenotype-based approach and GP indicated a small but significant discrepancy in the classifications. Wheeler et al. (2001, their Fig. 12a) and Kjer (2004) constructed phylogenetic trees of insects using 18S rDNA sequencing data and they employed information provided by phenotypic traits to optimize the final sequence-based phylogenetic tree and, thereby, to obtain a good match with the classical phylogenetic tree based only on phenotypic traits [10]. The trees they describe are, to some extent, similar to those obtained here (Fig. S3). It should be noted that both phenotype-based and 18S rDNA based classification systems involve arbitrary elements such as selection of phenotypic traits and choice of analytical parameters even though they are defined systematically. This inherent characteristic must govern the final shape of the phylogenetic trees. To our merit, the GP-based approach requires only one special parameter that determines the relative weight of the temperature and mobility and is empirically fixed [26]. Nevertheless, the fact that such different and independent approaches, namely, phenotype-based and GP-based, generated congruent classifications is a surprise and provides us with a challenge of explaining this congruency since there was no a *priori* expectation of this outcome. At present, we are unable to explain the congruency but can only leave this matter open for speculation by those interested in biological classification. In conclusion, GP provides a robust and relatively simple means of identifying and classifying insects and other organisms in general, and is probably a more effective approach for preliminary phylogenetic tree construction than 18S rDNA sequencing.

## Materials and Methods

### A. Genome Profiling (GP)

Preparation of DNA is carried out by the alkaline extraction method [27]. Briefly, the procedures adopted are as follows: 1) An aliquot containing cells was transferred into an Eppendorf tube; 2) After adding 3 µl of 0.5 M NaOH, the sample solution was incubated at 94°C for 5 min and then at 64°C for 60 min; 3) the sample solution was neutralized with 5 µl of 200 mM Tris-HCl (pH 8.0) buffer, and incubated at 65°C.

GP contains two major experimental steps: random PCR and temperature gradient gel electrophoresis (TGGE) (The whole procedure is shown in Fig. S1). Random PCR is a process in which DNA fragments are sampled at random from genomic DNA through a mismatch-containing hybridization of a primer to a template DNA during PCR [27]. Random PCR can be performed using a single primer of dodeca-nucleotides (pfM12, dAGAACGCGCCTG) with the 5′-end Cy3-labeled. This primer sequence has been recommended for general use including the application to animal cells [16]. The PCR reaction (50 µl) usually contains 200 µM dNTPs (N = G,A,T,C), 0.5 µM primer, 10 mM Tris-HCl (pH 9.0), 50 mM KCl, 2.5 mM MgCl_2_, 0.02 unit/µl Taq DNA polymerase (Takara Bio, Shiga, Japan) and a particular amount of template DNA. Random PCR was carried out with 30 cycles of denaturation (94°C, 30 s), annealing (26°C, 2 min) and extension (47°C, 2 min) using e.g., a PTC-100TM PCR machine (MJ Research, Inc., Massachusetts, USA). The DNA samples were subjected to μ-TGGE [28], which adopts a tiny slab gel of 24×16×1 mm^3^ for electrophoresis using a temperature-gradient generator, μ-TG (Taitec, Saitama, Japan). In each run of electrophoresis, an internal reference DNA is co-migrated. The 200-bp reference DNA (the 191-bp bacteriophage fd gene VIII, sites 1350∼1540 attached to a 9-bp sequence, CTACGTCTC, at the 3′-end) is experimentally determined to have a melting temperature of 60°C under standard conditions. The gel used was composed of 6% acrylamide (acrylamide∶bis = 19∶1) containing 90 mM Tris-HCl (pH 8.0), 90 mM boric acid, 2 mM EDTA and 8 M urea. The linear temperature gradient was run from 15°C to 65°C. The loading amount of amplified DNA was around 2 µg, which was subjected to this temperature gradient gel electrophoresis for 12 minutes at 5 V/cm. After electrophoresis, DNA bands were detected with a fluorescence imager, Molecular Imager FX (Biorad, Hercules, CA) or by silver staining [29].

### B. Obtaining spiddos, PaSS, and genome distance

Genome profiling data obtained by the GP technology are highly informative but difficult to manage due to their complexity. However, this inconvenience could be overcome by introducing spiddos (species identification dots) derived from featuring points [26]. The featuring points correspond to those where structural transitions of DNA occur, such as double-stranded to single-stranded DNA [19], [30]. A set of spiddos can be used to provide a sufficient amount of information for identifying species [26]. Using spiddos, we can define the **pattern similarity score** (*PaSS*) between two genomes as follows:
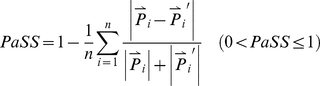
(1)where 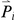 and 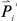 correspond to the normalized positional vectors (composed of two elements, mobility (*μ*) and temperature (θ) in Fig. 1) for spiddos *P_i_* and *P_i′_* collected from two genome profiles (discriminated with or without a prime), respectively, and *i* denotes the serial number of spiddos (supplementary comment: If the two species are sufficiently close, the assignment of the corresponding feature points is self-evident. However, as they get to be more distant it becomes more and more probabilistic to assign the corresponding feature points. Therefore, we have introduced a general definition for the PaSS value: The PaSS value between two species is assumed to be the maximum value obtained after the computer-aided exhaustive combinations of a set of spiddos between two organisms. The effectiveness of this approach has been experimentally supported [17], [18] and theoretically considered [26]). A database site has been constructed [23] in order to provide semi-automatic data processing [22]. The PaSS value thus introduced is empirically known to be a good measure to quantify the closeness or the distance between two species (or cells) [26]. In short, PaSS provides a measure how two set of sppidos can be closely superposed, generating a higher value (maximum: 1) when they are more closely related mutually. The genome distance d_G_ is conveniently defined here as 1−PaSS [17].

### C. Cluster Analysis for GP data

To cluster species based on genome distance (d_G_), a clustering software, FreeLighter [18] was developed based on Ward's method, a type of nearest neighbor method [31], [32]. This method is based on the distance defined in Eq. 2 which implies that Clusters *a* and *b* are to be merged into *c*, and *x* is an arbitrary cluster:
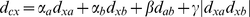
(2)where *α_a_*, *α_b_*, *β*, and *γ* are weighing parameters, *d_xa_*, *d_xb_*, *d_ab_*, and *d_cx_* represent distances between relevant clusters such as Cluster *x* and Cluster *a* for *d_xa_*. Briefly, the distance between a particular element or cluster (x) and a cluster synthesized from clusters a and b can be defined in Eq. 2, which is progressively iterated.

### D. 18S rDNA sequencing and cluster analysis

DNA molecules for 18S rRNA were PCR amplified using our newly designed primers of the sequences 5′GGCCGGTACGTTTACTTTGA-3′ (for forward) and 5′ CAATCCCTAGCACGAAGGAG-3′ (for reverse). Amplification conditions: 1 cycle-94°C (5 min); 30 cycles-94°C (30 sec.), 55°C (1 min), 72°C (1 min); 1 cycle-72°C (10 min). DNAs were cloned in pGEM-T Easy Vector using pGEM-T Easy Vector System (Promega). Sequences were determined for both strands and published from NCBI, EMBL, and DDBJ. Accession number of each species is shown in Table S4. ClustalW program was used to cluster 18S rDNA sequences of 26 species.

### E. Congruence value (*V_c_* and *V_c_*′)

We introduced this value to compare two trees (dendrographs) and to obtain the closeness of them quantitatively as described in detail in Text S1 (appendix paper). It provides a novel algorithm for scoring the similarity of two trees employing Cluster Matching Score (CMS), obtained by matching clusters between trees under some criteria. Then Congruence value (*Vc*) and modified congruence value (*Vc*′) are defined as follows:
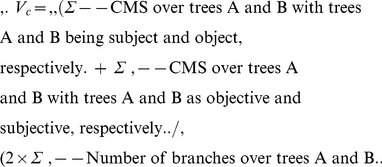
(3)where 0≤*V*_c_≤1, the terms ‘subject’ and ‘object’ are also defined in Text S1, and

*V_c_′* ∶ *V_c_* obtained after the coarse-graining of one partner of a pair of trees which is more finely structured. This can be done by bunching level different clusters under a bunching criterion such as compression of less than 15% height difference.

## Supporting Information

Figure S1 **The procedure used to identify species by GP.** During random PCR, primer binding occurs in a mismatch-containing structure due to the relaxed mode of PCR, thus enabling us to sample DNA fragments from various sites of the genomic DNA just like random-sampling in statistics. In TGGE, DNA fragments layered on the top of a slab gel migrate downward with drawing a characteristic curvature caused by the temperature gradient. Featuring point(s) of each DNA fragment is/are assigned and processed to generate *species identification dots* (*spiddos*) with a computer. The *PaSS* (pattern similarity score) calculation is performed as described in Equation 1 in methods of this supporting materials. This figure was taken from *BMC Genomics* (Ref. 17, with slight modifications).
(TIF)

Figure S2 **Phylodendrons of plants (A1∼A12), insects (B1∼B14), and fish (C1∼C14).** Only 3 of the insects dealt here (14 species) are also used in the present study (3 out of 26 species). Phenotypic (left) and genotypic (right) trees are drawn on the basis of taxonomic hierarchy or PaSS value, respectively. The nomenclatures of these organisms are appearing in Supplementary table S1 (Ref. 18). Photographs (far left) and *spiddos* (far right) are included to illustrate the technique. Trees were drawn by the *group average method* (plants) or the *median method* (insects and fish) using a cluster program (*FreeLighter*) (Ref. 18). This figure was taken from Ref. 18, *International Journal of Plant Genomics*, which can be freely distributed.
(TIF)

Figure S3 **Comparison between phylogenetic tree topologies (modified from** **Fig. 3** **of the present study and** **Fig. 1** **of Ref. 9) based on hierarchy of insects Order.** A) phenotype-based one presented by Iwatsuki *et al.*, (1960). B) GP-based. C) 18S-rDNA sequence-based one presented in Ref. 9.
(TIF)

Text S1 **Congruence value (** ***V_c_*** **): A measure to evaluate the similarity between two (phylogenetic) trees.**
(PDF)

Table S1 **Taxonomy^†^ of the species dealt in this study.**
(DOC)

Table S2 **Tentative comparison in terms of cost, labor and other consumables between 18S rDNA sequencing and GP experiments.**
(DOC)

Table S3 **The basic data for tentative estimation of experimental cost in Yen (Japan, 2009).**
(DOC)

Table S4 **Genome sources.**
(DOC)
