# Supplementary figures and images for: Genome Profiling (GP) Method Based Classification of Insects: Congruence with That of Classical Phenotype-Based One
Source: PLoS One. 2011 Aug 31;6(8):e23963. doi: 10.1371/journal.pone.0023963 (PMC3166070; doi:10.1371/journal.pone.0023963)

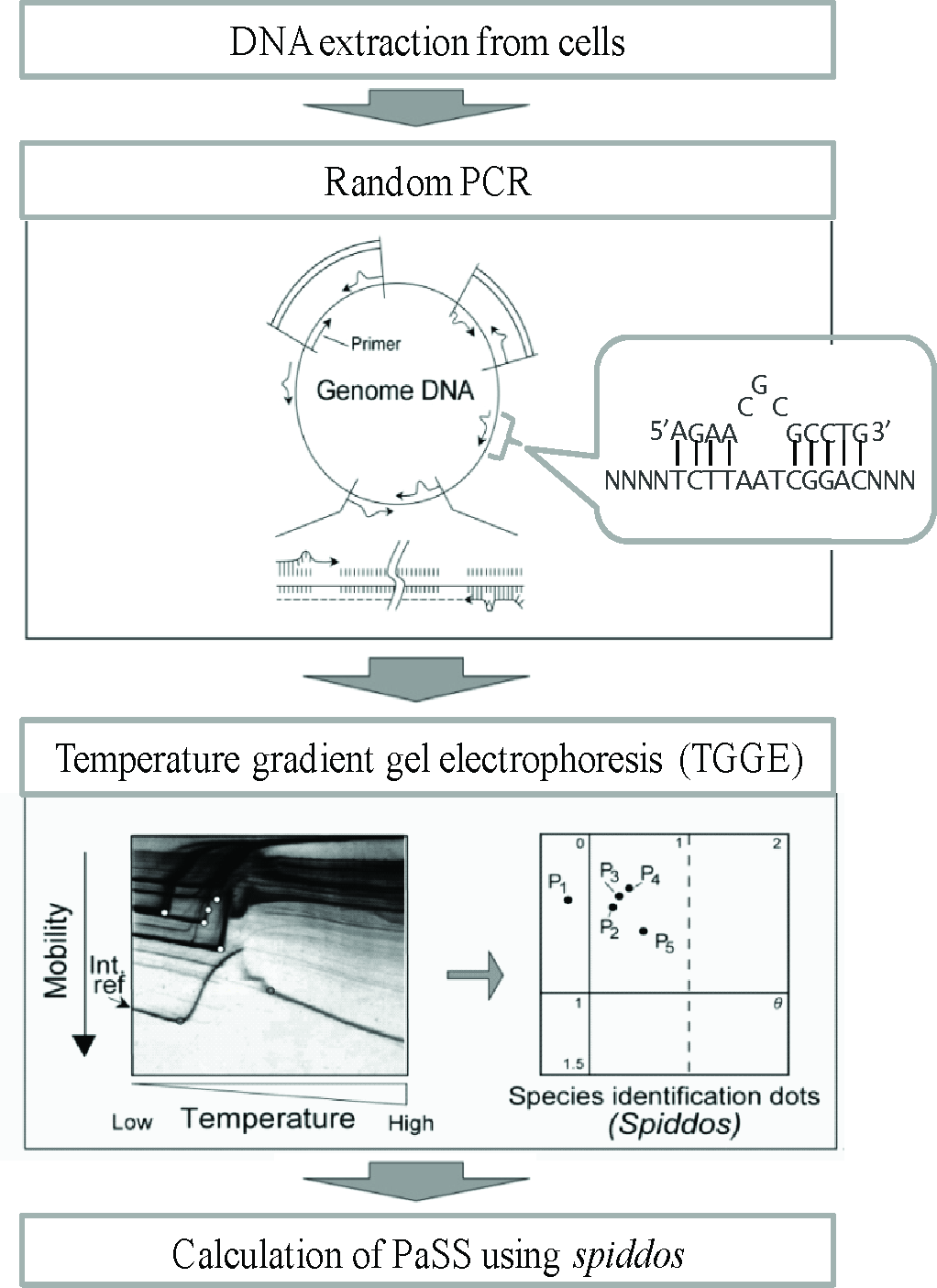

Supplement: Figure S1 — The procedure used to identify species by GP. During random PCR, primer binding occurs in a mismatch-containing structure due to the relaxed mode of PCR, thus enabling us to sample DNA fragments from various sites of the genomic DNA just like random-sampling in statistics. In TGGE, DNA fragments layered on the top of a slab gel migrate downward with drawing a characteristic curvature caused by the temperature gradient. Featuring point(s) of each DNA fragment is/are assigned and processed to generate species identification dots (spiddos) with a computer. The PaSS (pattern similarity score) calculation is performed as described in Equation 1 in methods of this supporting materials. This figure was taken from BMC Genomics (Ref. 17, with slight modifications). (TIF) [file pone.0023963.s001.tif]

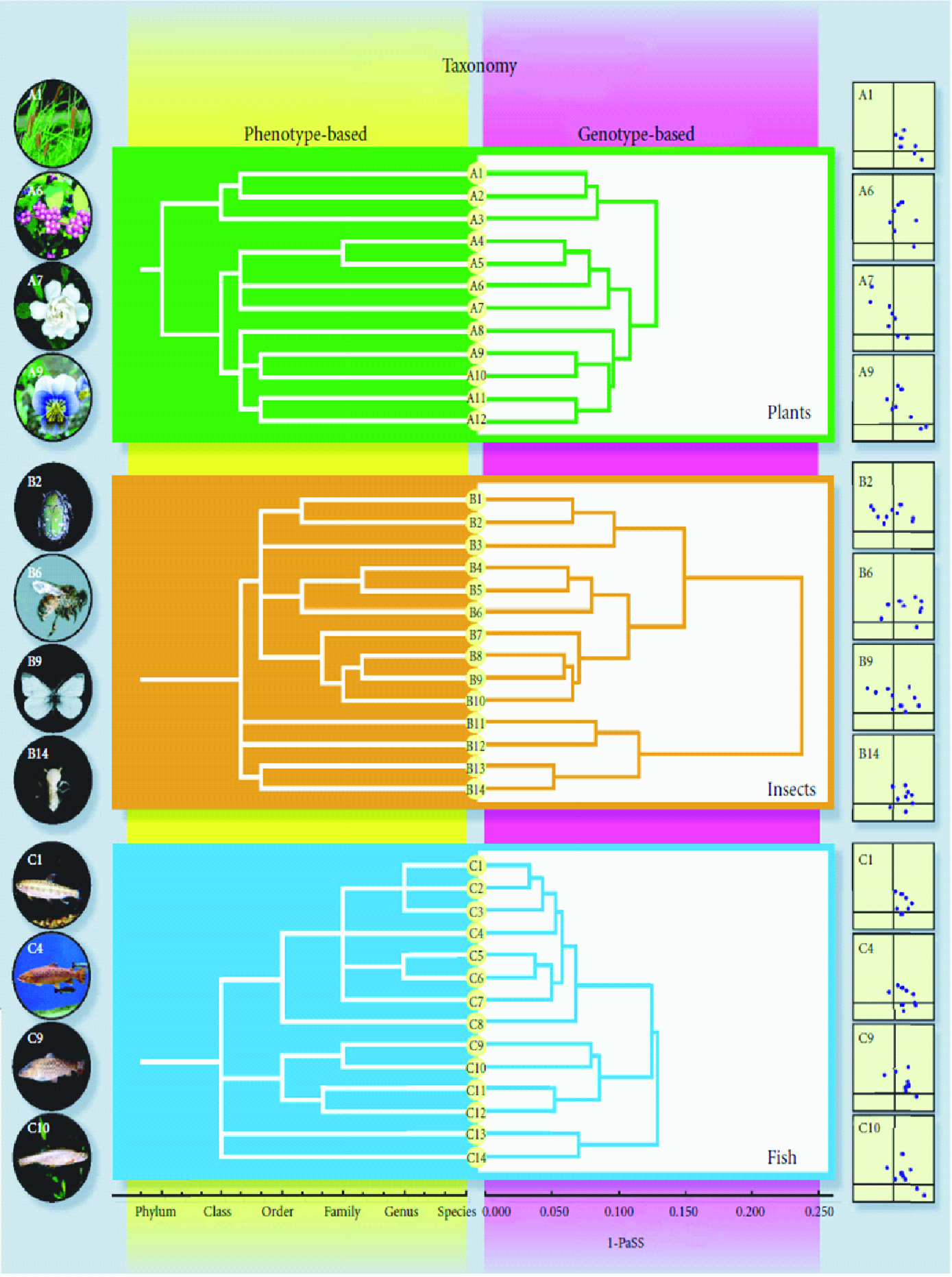

Supplement: Figure S2 — Phylodendrons of plants (A1∼A12), insects (B1∼B14), and fish (C1∼C14). Only 3 of the insects dealt here (14 species) are also used in the present study (3 out of 26 species). Phenotypic (left) and genotypic (right) trees are drawn on the basis of taxonomic hierarchy or PaSS value, respectively. The nomenclatures of these organisms are appearing in Supplementary table S1 (Ref. 18). Photographs (far left) and spiddos (far right) are included to illustrate the technique. Trees were drawn by the group average method (plants) or the median method (insects and fish) using a cluster program (FreeLighter) (Ref. 18). This figure was taken from Ref. 18, International Journal of Plant Genomics, which can be freely distributed. (TIF) [file pone.0023963.s002.tif]

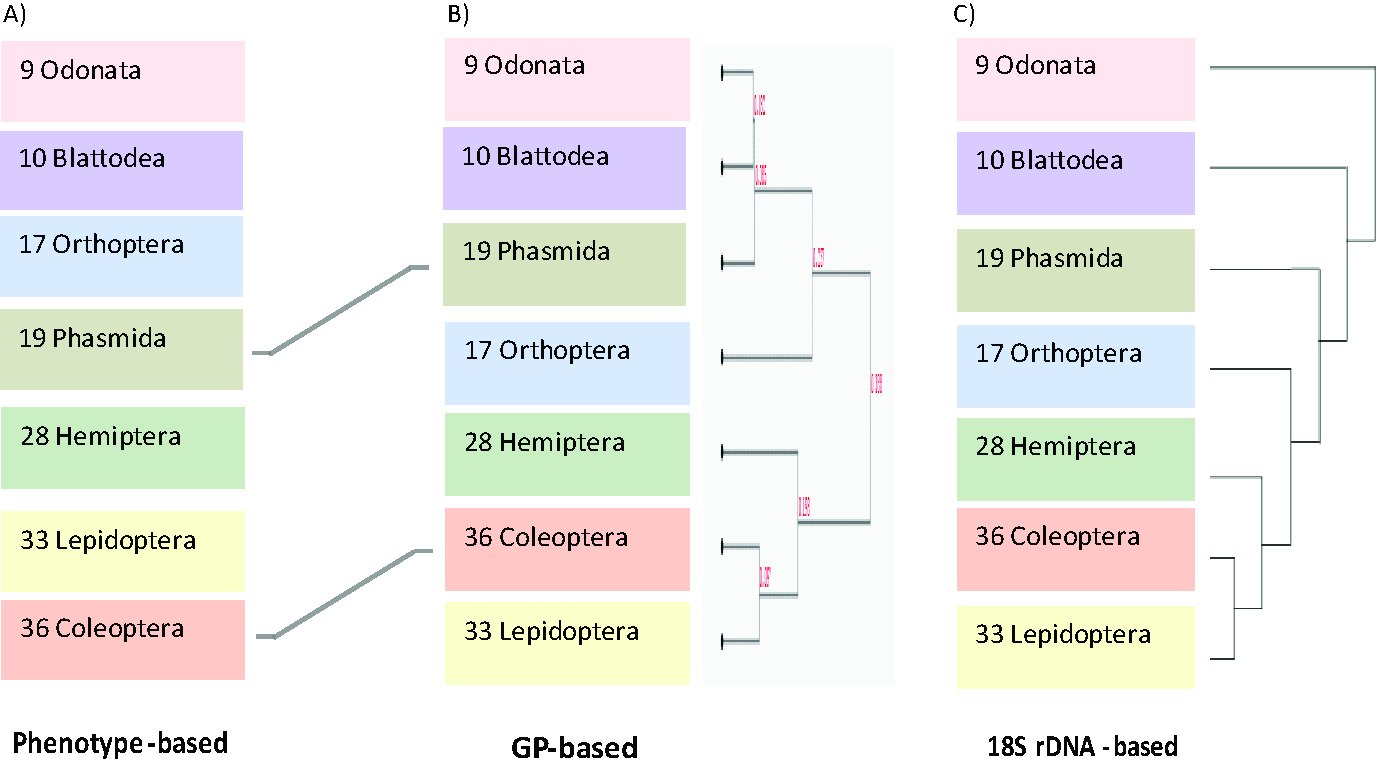

Supplement: Figure S3 — Comparison between phylogenetic tree topologies (modified from Fig. 3 of the present study and Fig. 1 of Ref. 9) based on hierarchy of insects Order. A) phenotype-based one presented by Iwatsuki et al., (1960). B) GP-based. C) 18S-rDNA sequence-based one presented in Ref. 9. (TIF) [file pone.0023963.s003.tif]
