# Supplementary material for: Genome Profiling (GP) Method Based Classification of Insects: Congruence with That of Classical Phenotype-Based One
Source: PLoS One. 2011 Aug 31;6(8):e23963. doi: 10.1371/journal.pone.0023963 (PMC3166070; doi:10.1371/journal.pone.0023963)
Supplement: Text S1 — Congruence value ( Vc ): A measure to evaluate the similarity between two (phylogenetic) trees. (PDF) [file pone.0023963.s004.pdf]

Text S1:

Congruence value (V_c): A measure to evaluate the similarity between two (phylogenetic) trees

Shamim Ahmed and Koichi Nishigaki

Calculation of the congruence value between tree-A and tree-B is described hereafter.

1. Definitions of terms used here

The following terms were defined to determine the congruence value between two trees.

- ***Element*:** Each species is considered as a single element (SE) in this study. When couples of single elements formed a cluster (group), they are termed as cluster-elements (CE)
- ***Cluster*:** is a term given to a set of elements stemmed from a common root. A cluster can be composed of sub-clusters and single elements.
- ***Level*:** A tree consists of different hierarchies of branches beginning from the bottom to the top level. The top level is identical to so-called "root". In the definition adopted here, "level" is used to express the hierarchy of each element or cluster. A cluster positioned at level ℓ always contains one or more cluster(s) positioned at level ℓ-1 by definition.
- ***Sub-cluster*:** A subset of a cluster consisting of more than or equal to two elements. A cluster of n elements has various kinds of sub-clusters, say, if n = 5; 5 kinds of 4-membered sub-clusters (=$^5C_4$), 10 kinds of 3-membered sub-clusters (=$^5C_3$), and 10 kinds of 2-membered sub-clusters (=$^5C_2$).
- ***Cluster matching score (CMS)*:** A score to evaluate the congruence between two clusters, which take into account the number of corresponding elements and the level difference of cluster.
- ***Phylogenetic tree/Tree*:** is a branching diagram showing the relationships of similarities or differences among various biological species (or elements) based on phenotypic and/or genotypic characteristics.
- ***Subject tree and Object tree***: When the congruence value between two trees is to be calculated, the trees are allotted as a subject tree and object one for the sake of convenience. If the tree-A is considered as the subject, then the tree-B will be treated as the object and vice versa.

2. Procedures:

Assigning matching clusters: In this study we assigned matching clusters (or sub-clusters) for relevant trees with a common cluster name based on the following criteria.

Criterion 1. If all elements in a cluster belonging to the subject tree-A correspond to the elements of a particular cluster in the object tree, a same name is assigned to both of the clusters like C_X (see Panel 1b).

Criterion 2. If more than 50% elements of a cluster of the subject tree-A correspond to some of the elements of a single cluster belonging to the object tree B, a same yet discriminative name is assigned as C_X for the cluster of the subject tree and C_X' for the cluster belonging to the tree B.

Criterion 3. No name is given if ≤50% elements of a cluster of the subjective tree can find the corresponding elements in any cluster contained in the objective tree.

How to calculate cluster matching score (CMS):

For C_x cluster (criterion 1):

CMS is the number of elements at level ℓ-1 contained in C_x cluster which is at level ℓ. i.e.,

$$CMS_{Cx} = \text{Number of elements in } C_x \text{ cluster} \quad (1)$$

If the cluster of interest contains the lower level elements than level ℓ-1, then those elements are scored with the reduction rate γ;

$$\gamma = (1/2)^{(\ell-1)-\ell'} = (1/2)^{(\ell-\ell')-1} = (1/2)^{\Delta\ell-1}$$

where ℓ, ℓ' and $\Delta\ell$ represent the level for the cluster of interest (of which CMS is calculated), level of the lower hierarchy cluster/element and the level difference, respectively.
Therefore, in such case, CMS will be expressed as follows;

$$CMS_{Cx} = \Sigma \text{ each element in Cx cluster x } (1/2)^{\Delta\ell-1} \quad (2)$$

For C_x' cluster (criteria 2):

When a sub-cluster is involved, the following consideration is made to calculate the CMS:

As an *m*-membered sub-cluster within a cluster of *n* elements ($n \geq m$) can appear in the number of states *S* (only single and same level elements are considered);

$$S = {}^{n}C_{m} = \frac{n!}{m!(m-n)!}$$

One possible way to consider this number of degeneracy in the calculation of CMS of C_x' cluster is to divide by this number *S*:

$$CMS_{Cx'} = \frac{1}{S} \text{ x Number of elements of Cx' cluster} \quad (3)$$

When a higher level cluster is assigned as C_x' which is composed of single and/or cluster elements positioned at level ℓ-1 and/or lower than level ℓ-1, in such cases CMS of C_x' cluster will be expressed as follows;

$$CMS_{Cx'} = \Sigma \text{ each element in } C_x\text{' cluster x } (1/2)^{\Delta\ell-1} \quad (4)$$

Definition of *Branch length and number:* If a cluster positioned at level ℓ and contains elements with a length more than 1 (ℓ- (ℓ-1) = 1unit, the branch length corresponding to the difference in the level), then the length of that particular branch (>1 length, marked as red in Panel 1c) will be considered as;

$$\text{Branch length} = (1/2)^{\Delta\ell - 1} \ (\text{unit}), \qquad (5)$$

where $\Delta\ell$ represent the level difference of a branch. If the $\Delta\ell > 4$ of a branch, it has been ignored in this study for the convenience.

Definition of congruence value (V_c):

The definition of the congruence value is:

$$V_c = (\sum \text{CMS over trees A and B with trees A and B being subject and object, respectively} + \sum \text{CMS over trees A and B with trees A and B as objective and subjective, respectively})/(2 \text{ x } \sum \text{Number of branches over trees A and B}), \qquad (6)$$

where $0 \leq V_c \leq 1$ and

V_c' : V_c obtained after the coarse-graining of one partner of a pair of trees which is more finely structured. This can be done by bunching level different clusters under a bunching criterion such as compression of less than 15% height difference.

3. Case studies

3.1 Example 1 (pair of trees A and B):

Case *π* of Tree A as subject one;

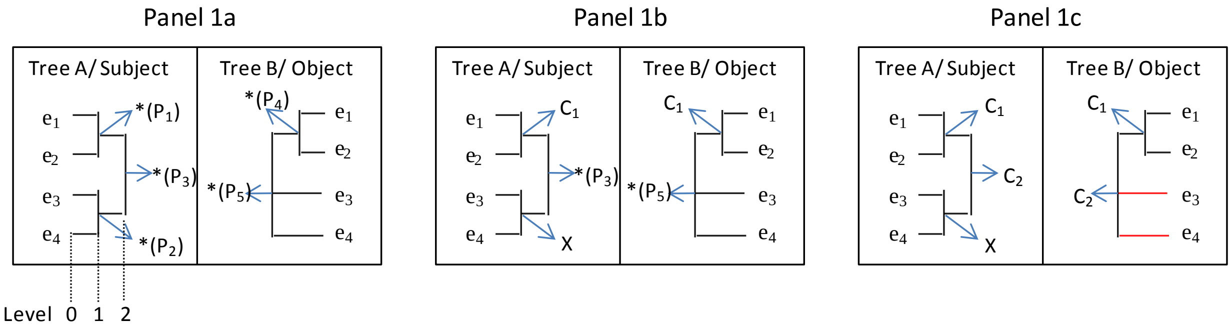

In Panel 1a, all of the possible clusters to be named are indicated with the star symbol (*).

First of all, the most basic level clusters of the subject tree A (i.e., P_1 and P_2) are subjected to the cluster matching examination against the clusters of the objective tree B. Since the possible clusters, P_1 and P_4, contain exactly the same elements (e_1 and e_2), they are assigned to be the congruent clusters and named as C_1 (Panel 1b). On the contrary, P_2 in the subject tree A cannot find a corresponding cluster in the objective tree B and thus cannot be named (denoted as X in Panel 1b). Now, we can see the trees with clusters named as in Panel 1b. Next level 2

cluster is dealt: i.e., P_3, which has the elements of e_3, e_4 and C_1 (cluster-element). When the possible cluster P_3 is compared with the clusters in the objective tree B, P_5 is an only possible candidate for the congruence cluster. Since the P_5 cluster has the same elements, i.e., e_3, e_4 and C_1, both P_3 and P_5 can be named with the same name, $C_{2,}$ as shown in Panel 1c.

Case (-*π*) of Tree B as subject one:

Panel 1d Panel 1e Panel 1f

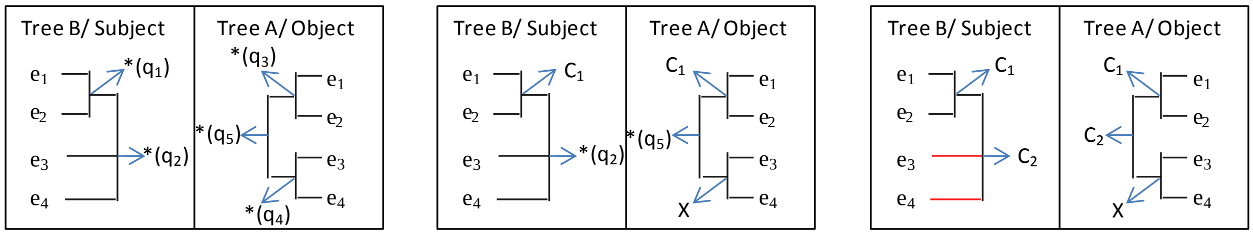

In the second session, the relationship of the subject and object is inversed for Trees A and B, rendering Tree A object and Tree B subject as in Panel 1d. Now, there are two (q_1 and q_2) and three (q_3~q_5) possible clusters in each tree.

Now, the most basic level cluster in the subjective tree B is q_1 only. The q_1 is examined to find its corresponding cluster in the objective tree A, providing with the corresponding one, i.e., q_3 and thus assigned as C_1 for both clusters.

Next, the higher level cluster q_2 (here, it is the top level cluster) is subjected to the same examination using Panel 1e. The cluster q_2 can find the corresponding matching cluster of q_5 which also contains the same elements of e_3, e_4 and C_1 as q_2 cluster does. Therefore, q_2 and q_5 can be named with the same name, $C_{2,}$ as shown in Panel 1f. Let's count V_c for all of the possible pairs.

CMS and the number of branches for the pair of Trees A and B:

For Case π (Panel 1c)

Tree name	Assigned cluster	Elements	CMS (Eq. adopted)	No. of branches
Tree A (subject)	C_1	e_1, e_2	2 (Eq. 1)	(1+1+1+1+1+1)
	C_2	C_1, e_3, e_4	(1+.5+.5) = 2 (Eq. 2)	= 6
Tree B (object)	C_1	e_1, e_2	2 (Eq. 1)	(1+1+1+.5+.5)
	C_2	C_1, e_3, e_4	2 (Eq. 2)	= 4
**Total**			**8**	**10**

For Case - π (Panel 1f)

Tree name	Assigned cluster	Elements	CMS (Eq. adopted)	No. of branches
Tree B (subject)	C_1	e_1, e_2	2 (Eq. 1)	(1+1+1+.5+.5)
	C_2	C_1, e_3, e_4	(1+.5+.5) = 2 (Eq. 2)	= 4
Tree A (object)	C_1	e_1, e_2	2 (Eq. 1)	(1+1+1+1+1+1)
	C_2	C_1, e_3, e_4	2 (Eq. 2)	= 6
**Total**			**8**	**10**

Congruence value (V_c):

$$V_c = (8+8)/(2 \times 10) = 16/20 = 0.8$$

3.2 Example 2 (pair of Tree C and Tree D):

Case π of Tree C as subject one;

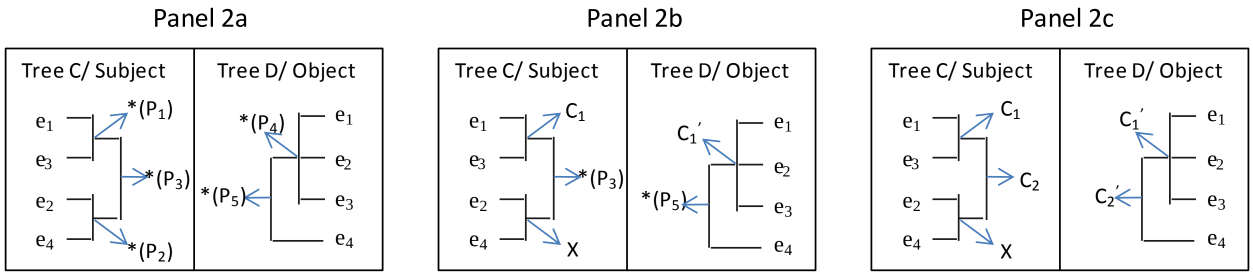

In Panel 2a, there are three (P_1~P_3) and two (P_4~P_5) possible corresponding clusters in this case.

In the first step, the most basic level clusters of the subject tree C (i.e., P_1 and P_2) are subjected to the cluster matching examination against the clusters of the objective tree D. P_1 of the subject tree contains two elements, e_1 and e_3, and P_4 of the object tree also contains e_1 and e_3 along with e_2 at the same cluster level. Therefore, P_1 & P_4 can be assigned as C_1 and C_1' based on criteria 2, respectively (Panel 2b). On the contrary, P_2 in the subject tree C cannot find a corresponding cluster in the objective tree D and thus cannot be named (denoted as X (extra naming) in Panel 2b).

The second step deals with the higher level cluster of subjective tree C, i.e., P_3, which has the elements of e_2, e_4 and C_1 (cluster-element). When the cluster P_3 is compared with the clusters in the objective tree D, P_5 is an only possible candidate for the congruence cluster. P_5 cluster is composed of elements, e_2, e_4 and C_1' and thus based on criteria 2, P_3 and P_5 can be named with the name C_2 and C_2', respectively as shown in Panel 2c.

Case ($-\pi$) of Tree D as subject one;

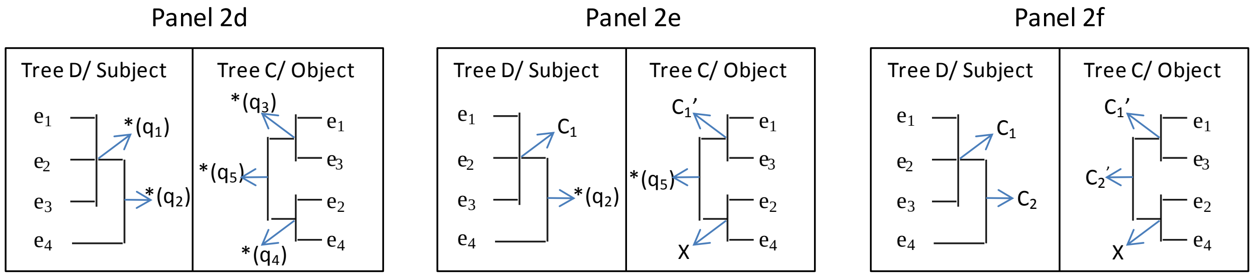

In the second session, the relationship of the subject and object is inversed, rendering the tree C object and the tree D subject as in Panel 2d. Now, there are two (q_1 and q_2) and three (q_3~q_5) possible clusters in each tree, respectively.

Then, the most basic level cluster in the subjective tree D is q_1 only, containing three elements, e_1, e_2 and e_3. The cluster q_1 is examined to find its corresponding cluster in the objective tree C and can find q_3 (containing two elements, e_1 and e_3), which is more than 50% of

the number of q_1 elements (three). Therefore, based on the assigning criteria 2, q_1 and q_3 can be assigned as C_1 and C_1', respectively (Panel 2e).
Next, the higher level cluster q_2 is subjected to the same examination using Panel 2e. The cluster q_2 (having the elements of C_1 and e_4) has a chance for finding the corresponding matching cluster of q_5 which contains the elements of e_2, e_4 and C_1'. In this case, e_4 representing as a common element for both trees and C_1' of object tree representing the more than 50% of C_1 cluster elements. Therefore, as a whole q_5 cluster has >50% elements of q_2 cluster of the subject tree and thus q_2 and q_5 can be assigned as C_2 and C_2', respectively (Panel 2f).

CMS and the number of branches for the pair of Trees C and D:

For Case π **(**Panel 2c)

Tree name	Assigned cluster	Elements	CMS (Eq. adopted)	No. of branches
Tree C (subject)	C_1	e_1, e_3	2 (Eq. 1)	(1+1+1+1+1+1) = 6
	C_2	C_1, e_2, e_4	(1+.5+.5) = 2 (Eq. 2)	
Tree D (object)	C_1'	2(e_1, e_3) of 3 (e_1, e_2, e_3)	2/3 (Eq. 3)	(1+1+1+1+.5) = 4.5
	C_2'	C_1', e_2, e_4	1+.5+.5 = 2 (Eq. 4)	
**Total**			**6.66**	**10.5**

For Case (-π) **(**Panel 2f)

Tree name	Assigned cluster	Elements	CMS (Eq. adopted)	No. of branches
Tree D (subject)	C_1	e_1, e_2, e_3	3 (Eq. 1)	(1+1+1+1+.5) = 4.5
	C_2	C_1, e_4	(1+.5) = 1.5 (Eq. 2)	
Tree C (object)	C_1'	2(e_1, e_3) of 3 (e_1, e_2, e_3)	0.66 (Eq. 3)	(1+1+1+1+1+1) = 6
	C_2'	C_1', e_2, e_4	1+.5+.5 = 2 (Eq. 2)	
**Total**			**7.16**	**10.5**

Congruence value (V_c):

$$\mathbf{V_c = (6.66 + 7.16)/(2 \times 10.5) = 13.82/20.5 = 0.65}$$

3.3 Example 3 (pair of Tree E and Tree F):

Case π of Tree E as subject one;

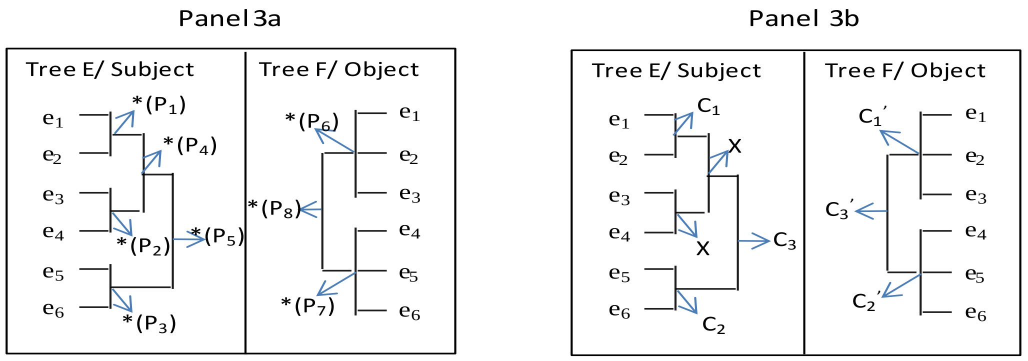

In panel 3a, there are five (P_1~P_5) and three (P_6~P_8) possible corresponding clusters in this case. Following the same algorithm as adopted in Cases 1 and 2, clusters C_1 ~ C_3 and C_1', C_2' and C_3' can be assigned as shown in Panel 3b.

Case (-π) of Tree F as subject one;

Panel 3c

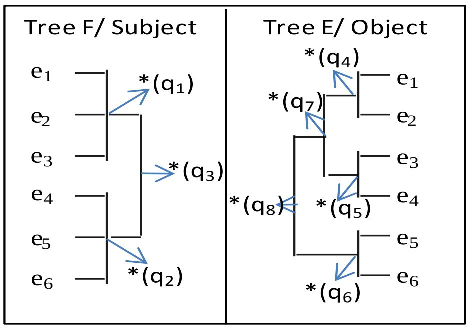

Panel 3d

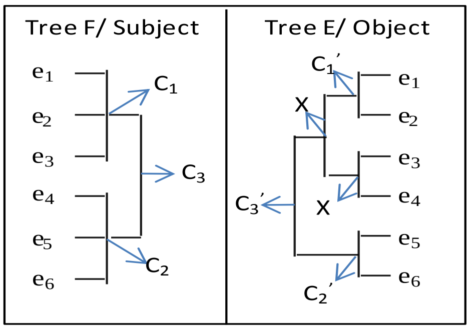

In the second session where the relationship of the subject and object is inversed, the named clusters C_1 ~ C_3 and C_1', C_2' and C_3' can be assigned as shown in Panel 3d.

CMS and the number of branches for the pair of Trees E and F:

For Case π (Panel 3b)

Tree name	Assigned cluster	Elements	CMS (Eq. adopted)	No. of branches
Tree E (subject)	C_1	e_1, e_2	2 (Eq. 1)	(1+1+1+1+1+1+1+1 +1+.5) = 9.5
	C_2	e_5, e_6	2 (Eq. 1)	
	C_3	C_1, e3, e_4, C_2	.5+.25+.25+.5 = 1.5 (Eq. 2)	
Tree F (object)	C_1'	2(e_1, e_2) of 3 (e_1, e_2, e_3)	2/3 (Eq. 3)	(1+1+1+1+1+1+1 +1) = 8
	C_2'	2(e_5, e_6) of 3 (e_4, e_5, e_6)	2/3 (Eq. 3)	
	C_3'	C_1', e_3, e_4, C_2'	1+.5+.5+1=2.5 (Eq. 4)	
**Total**			**9.32**	**17.5**

For Case (-π) (Panel 3d)

Tree name	Assigned cluster	Elements	CMS (Eq. adopted)	No. of branches
Tree F (subject)	C_1	e_1, e_2, e_3	3 (Eq. 1)	(1+1+1+1+1+1+1 +1) = 8
	C_2	e_4, e_5, e_6	3 (Eq. 1)	
	C_3	C_1, C_2	1+1 = 2 (Eq. 2)	
Tree E (object)	C_1'	2(e_1, e_2) of 3 (e_1, e_2, e_3)	2/3 (Eq. 3)	(1+1+1+1+1+1+1+1 +1+.5) = 9.5
	C_2'	2(e_5, e_6) of 3 (e_4, e_5, e_6)	2/3 (Eq. 3)	
	C_3'	C_1', e_3, e_4, C_2'	.5+.25+.25+.5= 1.5 (Eq. 4)	
**Total**			**10.82**	**17.5**

Congruence value (V_c):

$$\mathbf{V_c = (9.32 + 10.82)/(2 \times 17.5) = 20.14/35 = 0.57}$$

3.4 Example 4 (pair of Tree G and Tree H):

Case π of Tree G as subject one; **Case ($-\pi$) of Tree H as subject one;**

Panel 4a

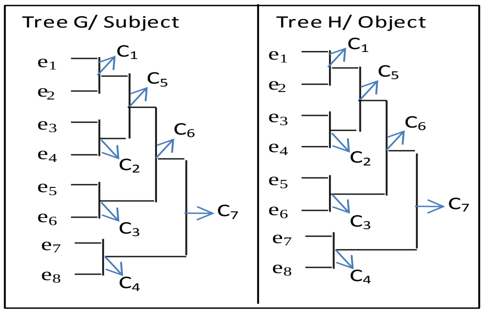

Panel 4b

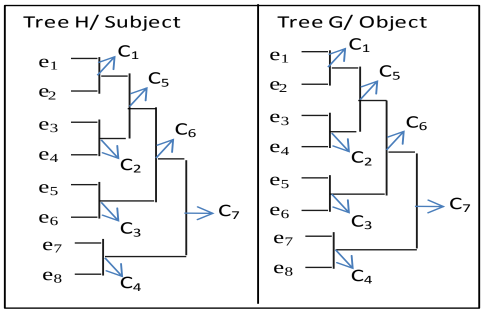

CMS and the number of branches for the pair of Trees G and H:

For Case π (Panel 4a)

Tree name	Assigned cluster	Elements	CMS (Eq. adopted)	No. of branches
Tree G (subject)	C_1	e_1, e_2	2 (Eq. 1)	(1+1+1+1+1+1+1+1+1 +1+1+1+.5+.25) = 12.75
	C_2	e_3, e_4	2 (Eq. 1)	
	C_3	e_5, e_6	2 (Eq. 1)	
	C_4	e_7, e_8	2 (Eq. 1)	
	C_5	C_1, C_2	2 (Eq. 2)	
	C_6	C_5, C_3	1+.5 = 1.5 (Eq. 2)	
	C_7	C_6, C_4	1+.25 = 1.25 (Eq. 2)	
Tree H (object)	C_1	e_1, e_2	2 (Eq. 1)	(1+1+1+1+1+1+1+1+1 +1+1+1+.5+.25) = 12.75
	C_2	e_3, e_4	2 (Eq. 1)	
	C_3	e_5, e_6	2 (Eq. 1)	
	C_4	e_7, e_8	2 (Eq. 1)	
	C_5	C_1, C_2	2 (Eq. 2)	
	C_6	C_5, C_3	1+.5 = 1.5 (Eq. 2)	
	C_7	C_6, C_4	1+.25 = 1.25 (Eq. 2)	
**Total**			**25.5**	**25.5**

For Case ($-\pi$) (Panel 4b)

Tree name	Assigned cluster	Elements	CMS (Eq. adopted)	No. of branches
Tree H (subject)	C_1	e_1, e_2	2 (Eq. 1)	(1+1+1+1+1+1+1+1+1 +1+1+1+.5+.25) = 12.75
	C_2	e_3, e_4	2 (Eq. 1)	
	C_3	e_5, e_6	2 (Eq. 1)	
	C_4	e_7, e_8	2 (Eq. 1)	
	C_5	C_1, C_2	2 (Eq. 2)	
	C_6	C_5, C_3	1+.5 = 1.5 (Eq. 2)	
	C_7	C_6, C_4	1+.25 = 1.25 (Eq. 2)	
Tree G (object)	C_1	e_1, e_2	2 (Eq. 1)	(1+1+1+1+1+1+1+1+1 +1+1+1+.5+.25) = 12.75
	C_2	e_3, e_4	2 (Eq. 1)	
	C_3	e_5, e_6	2 (Eq. 1)	
	C_4	e_7, e_8	2 (Eq. 1)	
	C_5	C_1, C_2	2 (Eq. 2)	
	C_6	C_5, C_3	1+.5 = 1.5 (Eq. 2)	
	C_7	C_6, C_4	1+.25 = 1.25 (Eq. 2)	
**Total**			**25.5**	**25.5**

Congruence value (V_c): V_c = (25.5 + 25.5)/(2 x 25.5) = 1

3.5 Example 5 (pair of Tree I and Tree J):

Case π of Tree I as subject one; **Case ($-\pi$) of Tree J as subject one;**

Panel 5a

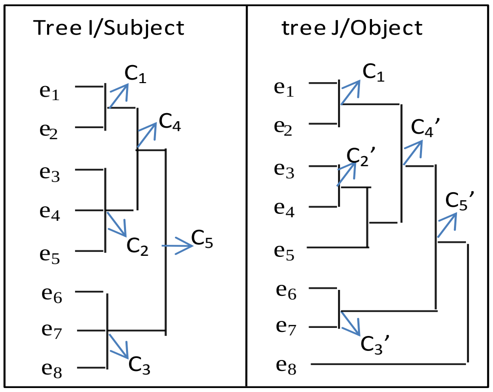

Panel 5b

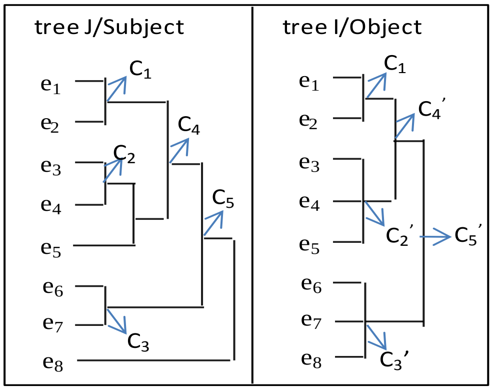

CMS and the number of branches for the pair of Trees I and J:

For Case π (Panel 5a)

Tree name	Assigned cluster	Elements	CMS (Eq. adopted)	No. of branches
Tree I (subject)	C_1	e_1, e_2	2 (Eq. 1)	(1+1+1+1+1+1+1+1+ 1+1+1+.5) = 11.5
	C_2	e_3, e_4, e_5	3 (Eq. 1)	
	C_3	e_6, e_7, e_8	3 (Eq. 1)	
	C_4	C_1, C_2	1+1=2 (Eq. 2)	
	C_5	C_4, C_3	1+.5=1.5 (Eq. 2)	
Tree J (object)	C_1	e_1, e_2	2 (Eq. 1)	(1+1+1+1+1+1+1+1+ 1+.5+.5+.25+.12) = 10.37
	C_2'	2(e_3, e_4) of 3 (e_3, e_4, e_5)	2/3 (Eq. 3)	
	C_3'	2(e_6, e_7) of 3 (e_6, e_7, e_8)	2/3 (Eq. 3)	
	C_4'	C_1, C_2', e_5	.5+.5+.25=1.25 (Eq. 4)	
	C_5'	C_4', C_3'	1+.25=1.25 (Eq. 4)	
**Total**			**17.32**	**21.87**

For Case ($-\pi$) (Panel 5b)

Tree name	Assigned cluster	Elements	CMS (Eq. adopted)	No. of branches
Tree J (subject)	C_1	e_1, e_2	2 (Eq. 1)	= 10.37
	C_2	e_3, e_4	2 (Eq. 1)	
	C_3	e_6, e_7	2 (Eq. 1)	
	C_4	C_1, C_2, e_5	.5+.5+.25=1.25 (Eq. 2)	
	C_5	C_4, C_3	1+.25=1.25 (Eq. 2)	
Tree I (object)	C_1	e_1, e_2	2 (Eq. 1)	= 11.5
	C_2'	2(e_3, e_4) of 3 (e_3, e_4, e_5)	2/3 (Eq. 3)	
	C_3'	2(e_6, e_7) of 3 (e_6, e_7, e_8)	2/3 (Eq. 3)	
	C_4'	C_1, C_2'	1+1=2 (Eq. 4)	
	C_5'	C_4', C_3'	1+.5=1.5 (Eq. 4)	
**Total**			**15.32**	**21.87**

Congruence value (V_c):

$$V_c = (17.32 + 15.32)/(2 \times 21.87) = 32.64/43.74 = 0.74$$

3.6 Example 6 (pair of Tree K and Tree L):

Case π of Tree K as subject one;

Panel 6a

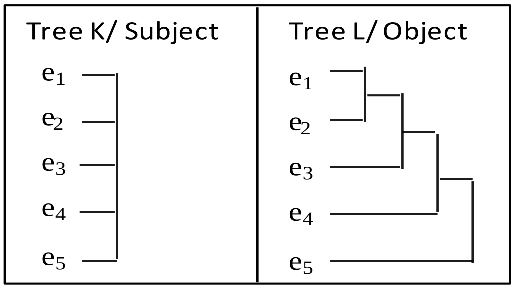

Case (-π) of Tree L as subject one;

Panel 6b

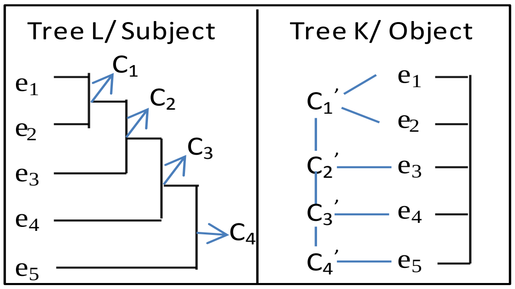

CMS and the number of branches for the pair of Trees K and L:

For Case π (Panel 6a)

Based on criterion 3, no name is given as ≤50% elements of the subjective tree found the corresponding cluster contained in the objective tree.

Tree name	Assigned cluster	Elements	CMS (Eq. adopted)	No. of branches
Tree K (subject)				(1+1+1+1+1) = 5
Tree L (object)				(1+1+1+1+1+.5+.25+.12) = 5.87
**Total**			**0.00**	**10.87**

For Case (-π) (Panel 6b)

Tree name	Assigned cluster	Elements	CMS (Eq. adopted)	No. of branches
Tree L (subject)	C_1	e_1, e_2	2 (Eq. 1)	= 5.87
	C_2	C_1, e_3	1+.5=1.5 (Eq. 2)	
	C_3	C_2, e_4	1+.25=1.25 (Eq. 2)	
	C_4	C_3, e_5	1+.12=1.12 (Eq. 2)	
Tree K (object)	C_1'	2(e_1, e_2) of 5 (e_1 ~ e_5)	.005 (Eq. 3)	= 5
	C_2'	2 (C_1', e_3) of 5	.005 (Eq. 3)	
	C_3'	2 (C_2', e_4) of 5	.005 (Eq. 3)	
	C_4'	2 (C_3', e_5) of 5	.005 (Eq. 3)	
**Total**			**5.89**	**10.87**

Congruence value (V_c):

$$V_c = (0.00 + 5.89)/(2 \times 10.87) = 5.89/21.74 = 0.27$$

4. Application to the current study

4.1 Congruence value (V_c) between phenotype and 18S rDNA-based (Fig. 2c) trees:

Case π of phenotype-based tree as subject one;

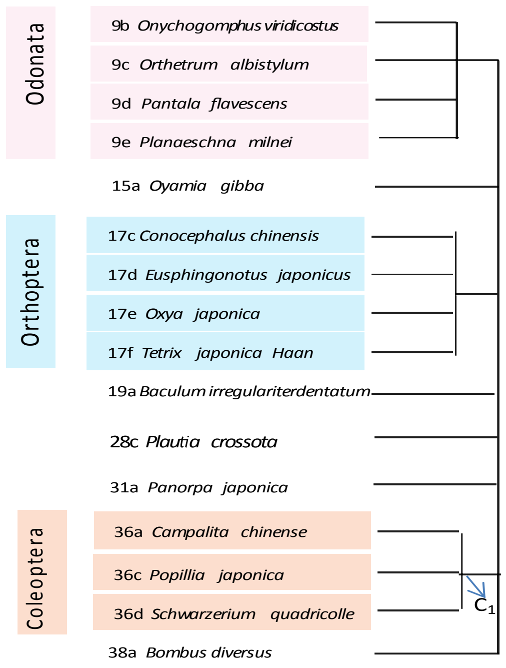

Phenotype-based/Subject tree

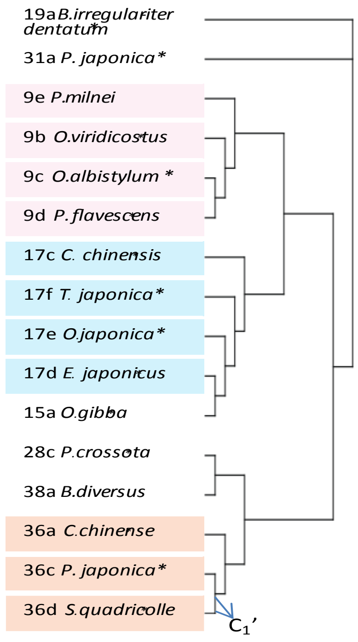

18S rDNA-based (Fig. 2c)/Object tree

Case (-π) of 18S rDNA-based as subject one;

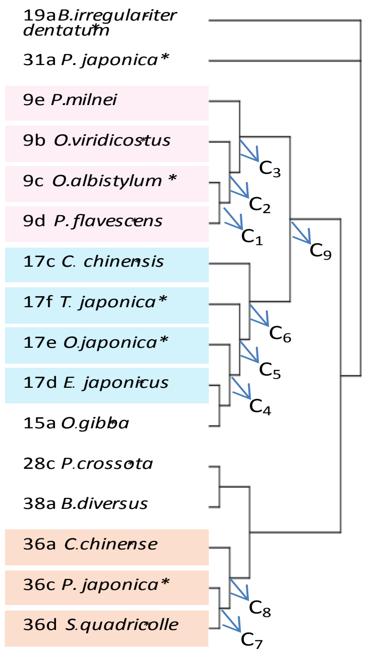

18S rDNA-based (Fig. 2c)/Subject tree

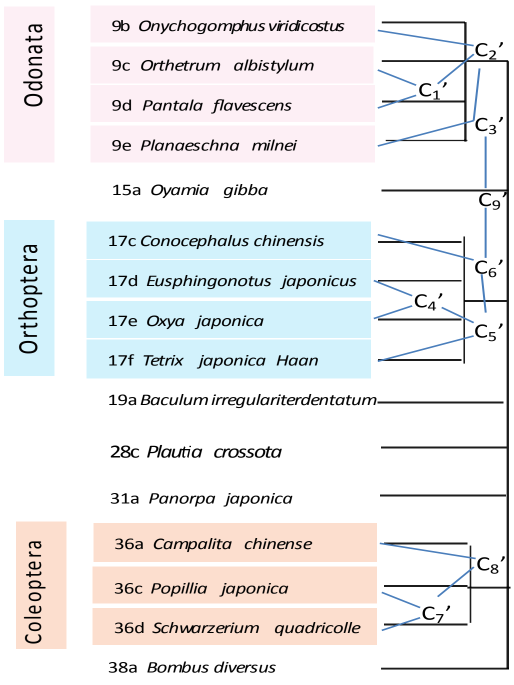

Phenotype-based/Object tree

CMS and the number of branches for the pair of phenotype and 18S rDNA-based (Fig. 2c) trees:

For Case π;

Tree name	Assigned cluster	Elements	CMS (Eq. adopted)	No. of branches
Phenotype-based tree (subject)	C_1	36a, 36c, 36d	3(Eq. 1)	=16.5
18S rDNA-based tree (object)	C_1’	2 (36c, 36d) of 3 (36 series)	2/3 (Eq. 3)	=17
**Total**			**3.66**	**33.5**

For Case ($-\pi$);

Tree name	Assigned cluster	Elements	CMS (Eq. adopted)	No. of branches
18S rDNA-based tree (Subject)	C_1	9c, 9d	2 (Eq. 1)	=17
	C_2	C_1, 9b	1+.5=1.5 (Eq. 2)	
	C_3	C_4, 9e	1+.25 = 1.25 (Eq. 2)	
	C_4	17e, 17d	.5+.5=1 (Eq. 2)	
	C_5	C_4, 17f	1+.25=1.25 (Eq. 2)	
	C_6	C_5, 17c	1+.12=1.12 (Eq. 2)	
	C_7	36c, 36d	2 (Eq. 1)	
	C_8	C_7, 36a	1+.5=1.5 (Eq. 2)	
	C_9	C_3, C_6	.25+.12=.37 (Eq. 2)	
Phenotype-based tree (Object)	C_1’	2 (9c, 9d) of 4 (9 series)	0.08 (Eq. 3)	=16.5
	C_2’	9b, C_1’	0.08 (Eq. 3)	
	C_3’	9e, C_2’	0.08 (Eq. 3)	
	C_4’	17d, 17e	0.08 (Eq. 3)	
	C_5’	17f, C_4’	0.08 (Eq. 3)	
	C_6’	17c, C_5’	0.08 (Eq. 3)	
	C_7’	36c, 36d	2/3 (Eq. 3)	
	C_8’	36a, C_7’	2/3 (Eq. 3)	
	C_9’	2 of 9 (9 &17series+15a)	<0.05 (Eq. 3)	
**Total**			**13.79**	**33.5**

Congruence value (V_c):

$$\mathbf{V_c = (3.66 + 13.79)/(2 \times 33.5) = 17.45/67 = 0.26}$$

4.2 Congruence value calculation between phenotype and 18S rDNA based (Fig. 2b) trees

Case π of phenotype-based tree as subject one;

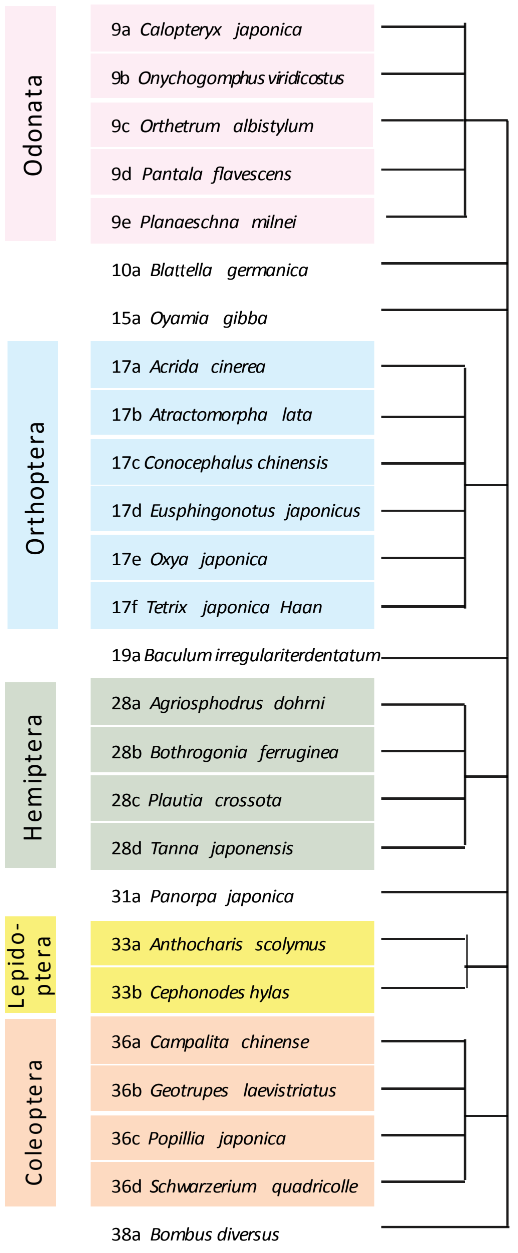

Phenotype-based/Subject tree

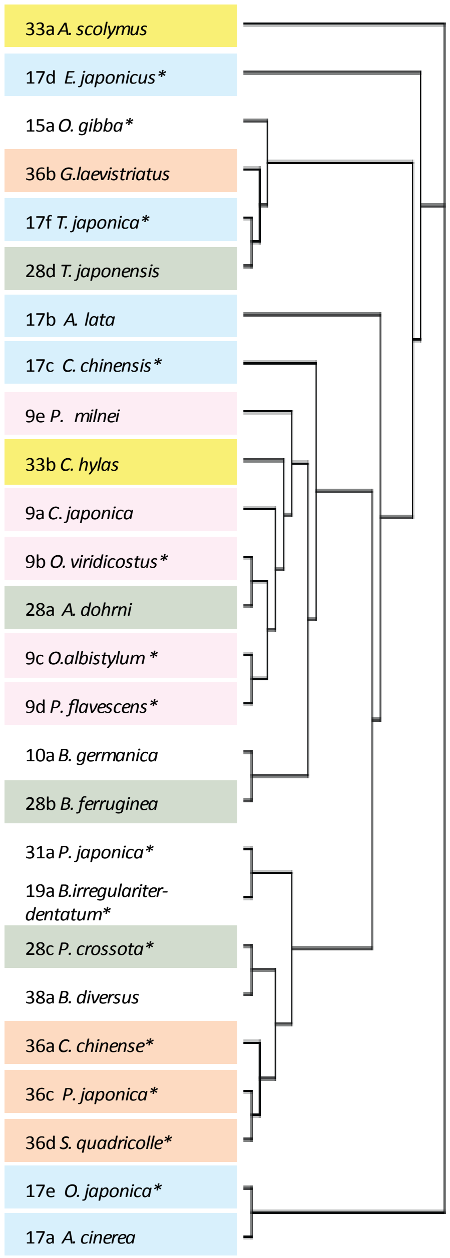

18S rDNA-based (Fig. 2b)/Object tree

Case (-π) of 18S rDNA-based as subject one;

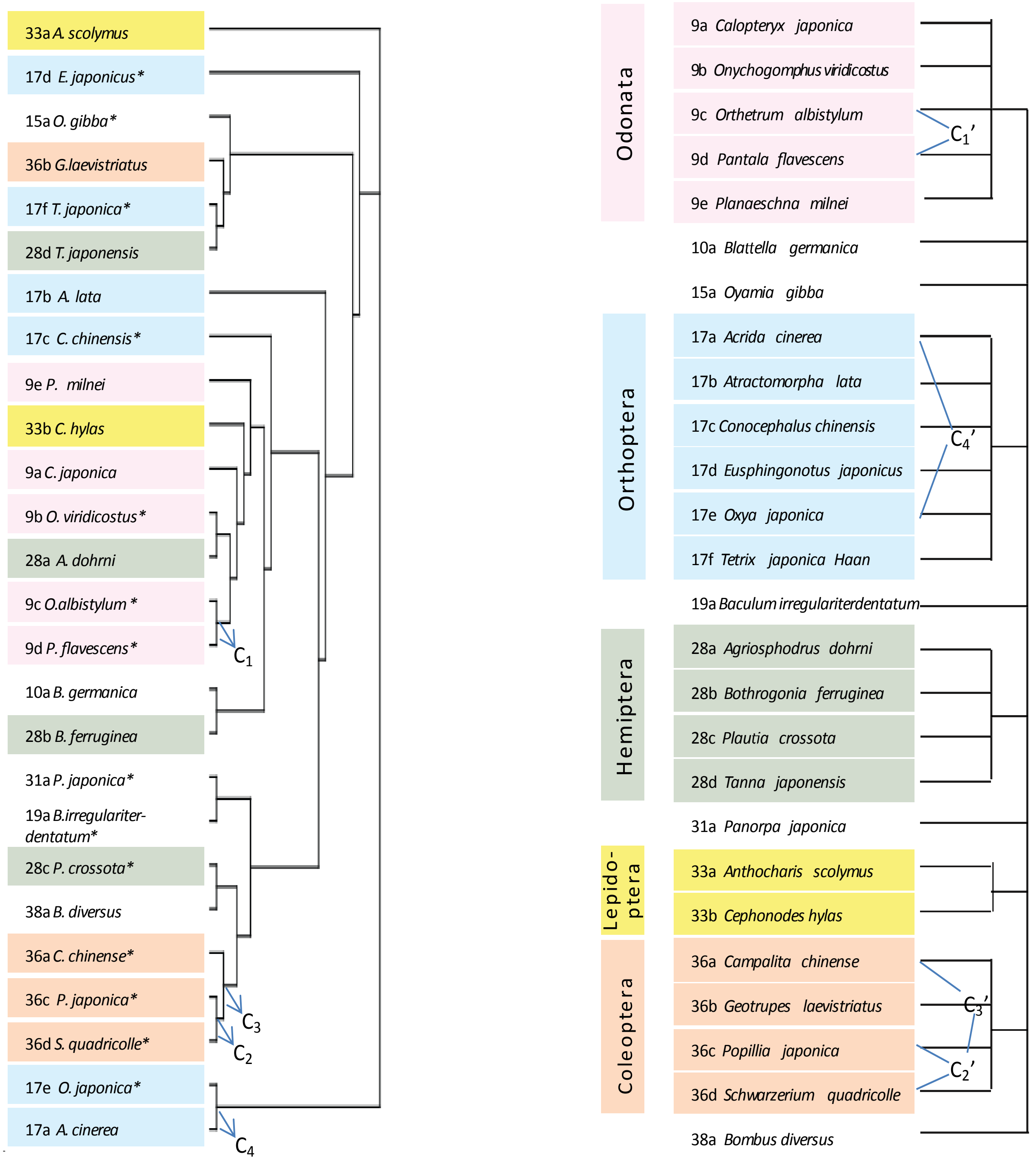

18S rDNA-based (Fig. 2b)/Subject tree | **Phenotype-based/Object tree**

Congruence value (V_c):

$$V_c = 0+7.5/(2 \times 58) = 0.06$$

4.3 Congruence value calculation between phenotype and GP-based (Fig. 3) trees.

Case π of Phenotype-based tree as subject one;

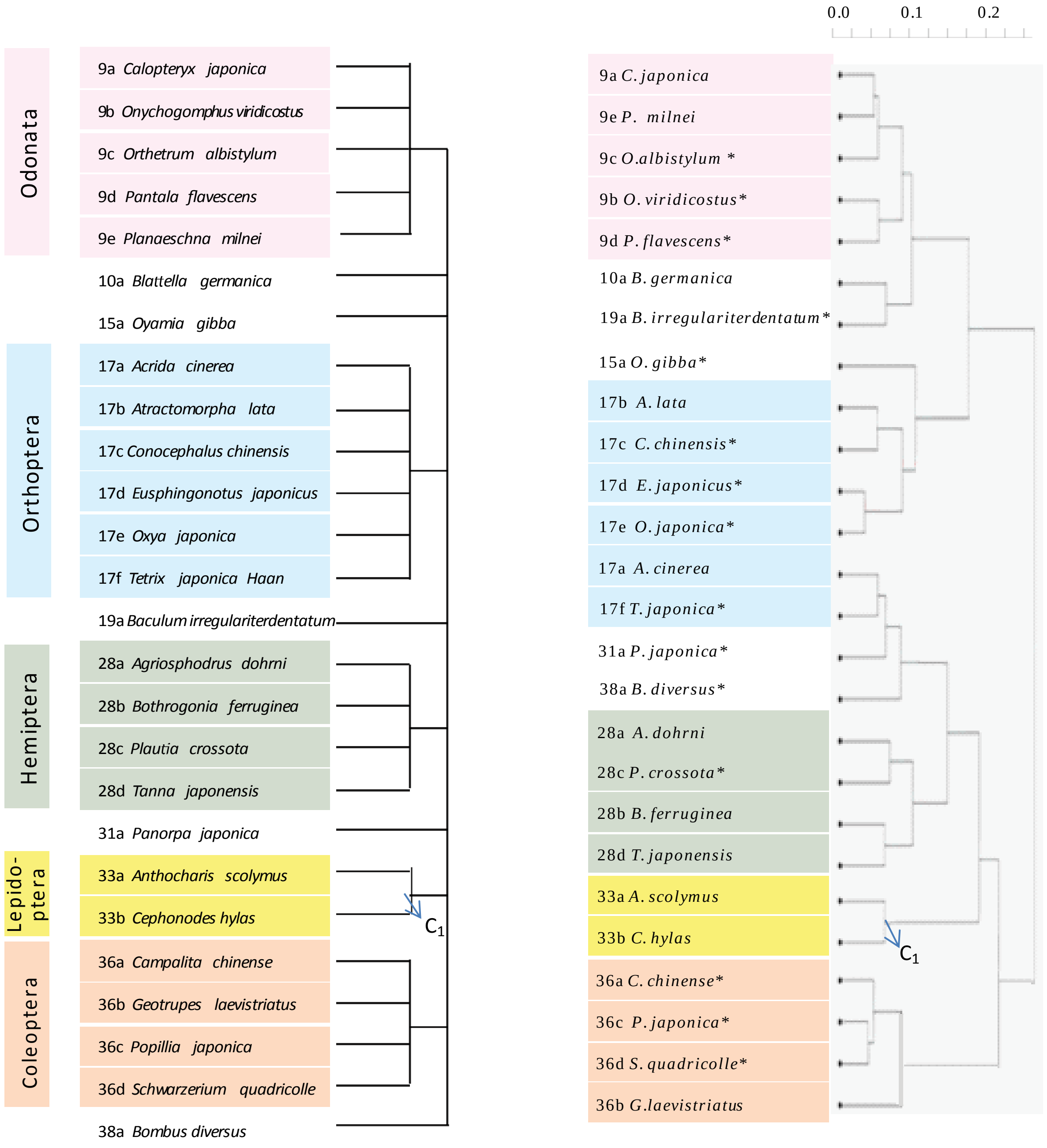

Phenotype-based/Subject tree **GP-based/Object tree**

Case (-π) of GP-based tree as subject one;

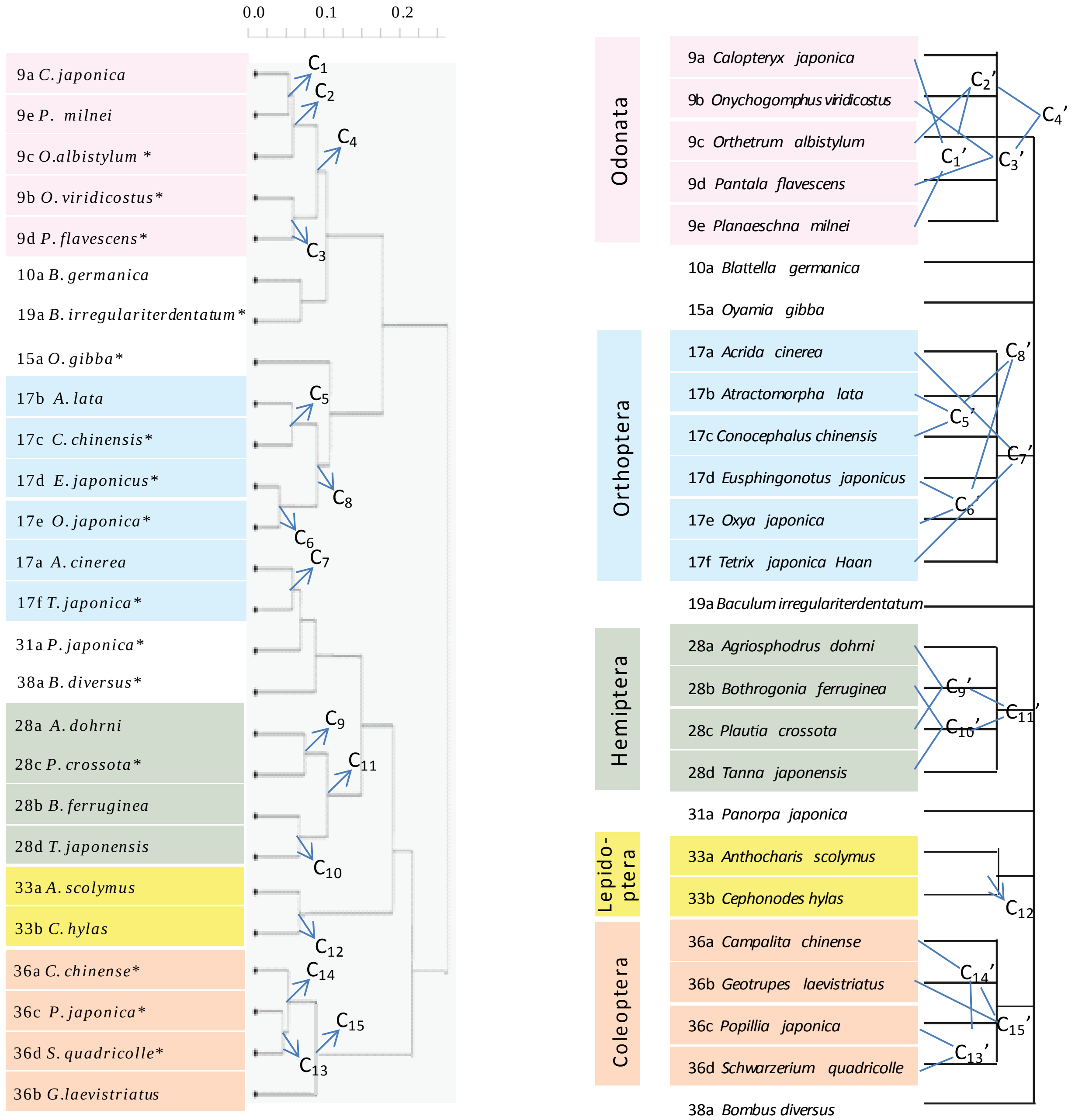

GP-based/Subject tree **Phenotype-based/Object tree**

CMS and the number of branches for the pair of phenotype and GP-based (Fig. 3) trees:

For Case π**;**

Tree name	Assigned cluster	Elements	CMS (Eq. adopted)	No. of branches
Phenotype-based tree (subject)	C_1	33a, 33b	2 (Eq. 1)	=28.5
GP-based tree (object)	C_1	33a, 33b	2 (Eq. 1)	=41.5
**Total**			**4**	**70**

For Case (-π)**;**

Tree name	Assigned cluster	Elements	CMS (Eq. adopted)	No. of branches
GP-based tree (Subject)	C_1	9a, 9e	2(Eq. 1)	=41.5
	C_2	9c, C_1	1.5(Eq. 1)	
	C_3	9b, 9d	2(Eq. 1)	
	C_4	C_2, C_3	2(Eq. 2)	
	C_5	17b, 17c	2(Eq. 1)	
	C_6	17d, 17e	2(Eq. 1)	
	C_7	17a, 17f	2(Eq. 1)	
	C_8	C_5, C_6	1+.5=1.5(Eq. 2)	
	C_9	28a, 28c	2(Eq. 1)	
	C_{10}	28b, 28d	2(Eq. 1)	
	C_{11}	C_9, C_{10}	2(Eq. 2)	
	C_{12}	33a, 33b	2(Eq. 1)	
	C_{13}	36c, 36d	2(Eq. 1)	
	C_{14}	36a, C_{13}	1.5(Eq. 2)	
	C_{15}	36b, C_{14}	1.25(Eq. 2)	
Phenotype-based tree (Object)	C_1’	2 (9a, 9e) 0f 5 (9 series)	<0.05(Eq. 3)	=28.5
	C_2’	9c, C_1’	<0.05(Eq. 4)	
	C_3’	2 (9b, 9d) 0f 5 (9 series)	<0.05(Eq. 3)	
	C_4’	C_2’, C_3’	<0.05(Eq. 3)	
	C_5’	2 (17b,17c) of 6 (17 series)	<0.05(Eq. 3)	
	C_6’	2 (17d,17e) of 6 (17 series)	<0.05(Eq. 3)	
	C_7’	2 (17a,17f) of 6 (17 series)	<0.05(Eq. 3)	
	C_8’	C_5’, C_6’	<0.05(Eq. 3)	
	C_9’	2(28a, 28c) of 4 (28 series)	<0.05(Eq. 3)	
	C_{10}’	2(28b, 28d) of 4 (28 series)	<0.05(Eq. 3)	
	C_{11}’	C_9’, C_{10}’	<0.05(Eq. 3)	
	C_{12}	33a, 33b	2(Eq. 1)	
	C_{13}’	2 (36c, 36d) of 4 (36 series)	<0.05(Eq. 3)	
	C_{14}’	36a, C_{13}’	<0.05(Eq. 3)	
	C_{15}’	36b, C_{14}’	<0.05(Eq. 3)	
**Total**			**29.75**	**70**

Congruence value (V_c):

$$V_c = (4 + 29.75)/(2 \times 70) = 33.75/140 = 0.24$$

4.4 Congruence value (V_c') between phenotype and 18S rDNA-based (Fig. 2c) trees after the coarse-graining process:

Case *π* of Phenotype-based tree as subject one;

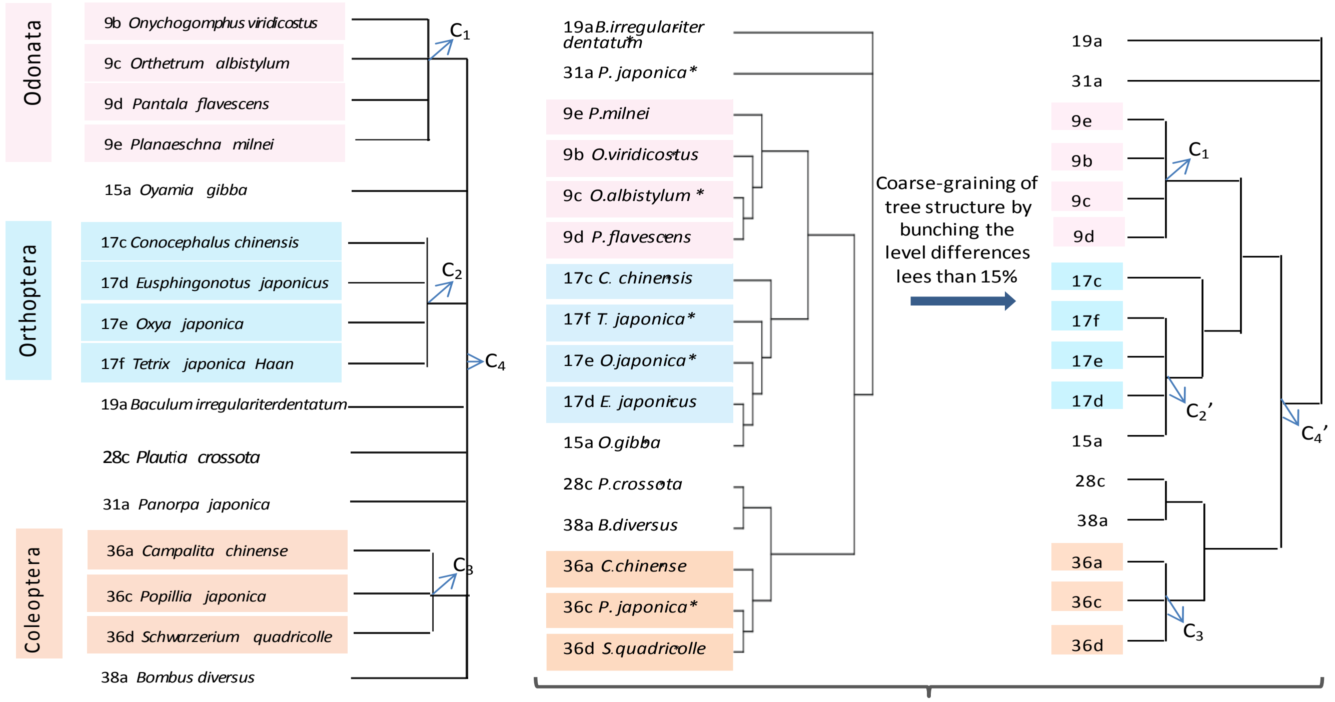

Phenotype-based/Subject tree **18S rDNA-based (Fig. 2c)/Object tree**

Case (-*π*) of 18S rDNA-based as subject one;

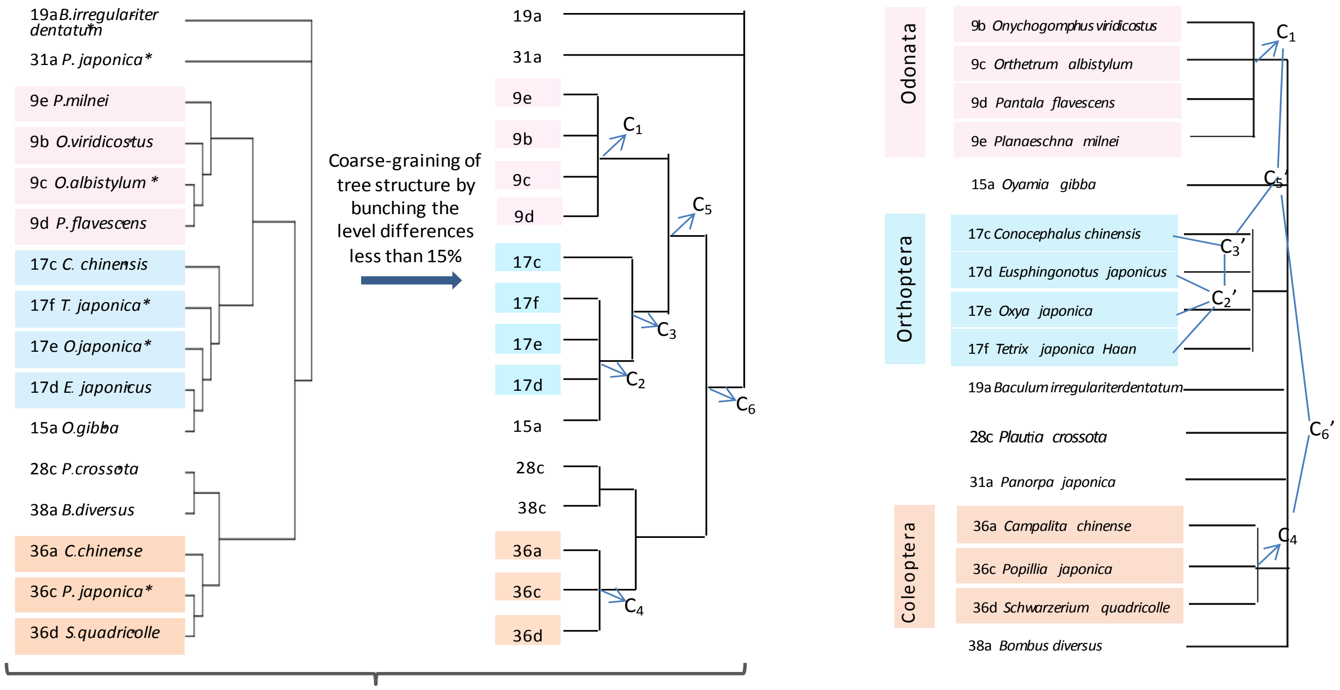

18S rDNA-based (Fig. 2c)/Subject tree **Phenotype-based/Object tree**

CMS and the number of branches for the pair of phenotype and 18S rDNA-based (Fig. 2c) trees:

For Case π**;**

Tree name	Assigned cluster	Elements	CMS (Eq. adopted)	No. of branches
Phenotype-based tree (Subject)	C_1	9b, 9c, 9d, 9e	4 (Eq. 1)	=16.5
	C_2	17c, 17d, 17e, 17f	4 (Eq. 1)	
	C_3	36a, 36c, 36d	3 (Eq. 1)	
	C_4	$C_{1,}$ $C_{2,}$ C_3, 15a, 19a, 28c, 31a, 28a	5.5 (Eq. 2)	
18S rDNA-based tree (Object)	C_1	9e, 9b, 9c, 9d	4 (Eq. 1)	=20.5
	C_2’	17f, 17e, 17d	¾= 0.75 (Eq. 3)	
	C_3	36a, 36c, 36d	3 (Eq. 1)	
	C_4’	$C_{1,}$ 17e, C_2’, 28c, 38a, C_3	.25+.12+.25+.12+.12+.25 =1.11	
**Total**			**25.36**	**37**

For Case (-π)**;**

Tree name	Assigned cluster	Elements	CMS (Eq. adopted)	No. of branches
18S rDNA-based tree (Subject)	C_1	9e, 9b, 9c, 9d	4 (Eq. 1)	=20.5
	C_2	17f, 17e, 17d, 15a	4 (Eq. 1)	
	C_3	$C_{2,}$ 17c	1+.5=1.5 (Eq. 2)	
	C_4	36a, 36c, 36d	4 (Eq. 1)	
	C_5	$C_{3,}$ C_1	1+.5=1.5 (Eq. 2)	
	C_6	$C_{5,}$ $C_{4,}$ 28c, 38a	1+.25+.12+.12=1.49	
Phenotype-based tree (Object)	C_1	9b, 9c, 9d, 9e	4 (Eq. 1)	=16.5
	C_2’	17f, 17e, 17d	¾= 0.75 (Eq. 3)	
	C_3’	C_2’, 17c	<0.05	
	C_4	36a, 36c, 36d	3 (Eq. 1)	
	C_5’	C_1, C_3’	1+1=2 (Eq. 4)	
	C_6’	C_5’, C_4, 19a, 28c, 31a	1+1+.5+.5+.5=3.5	
**Total**			**28.74**	**37**

Congruence value (V_c’):

$$V_c' = (25.36 + 28.74)/(2 \times 37) = 54/74 = 0.73$$

4.5 Congruence value (V_c') between phenotype and 18S rDNA-based (Fig. 2b) trees after the coarse-graining process:

Case π of Phenotype-based tree as subject one;

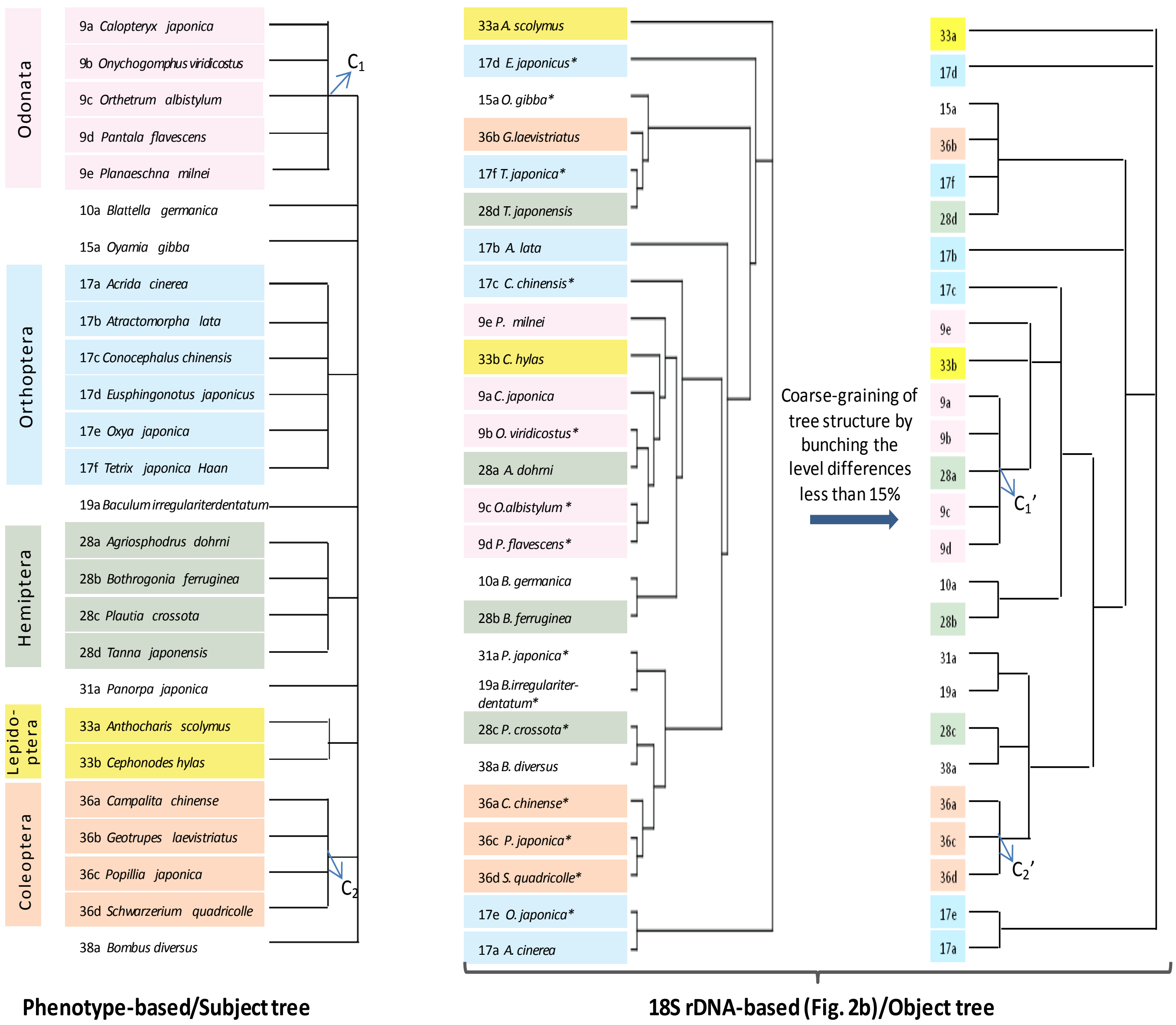

Case (-π) of 18S rDNA-based as subject one;

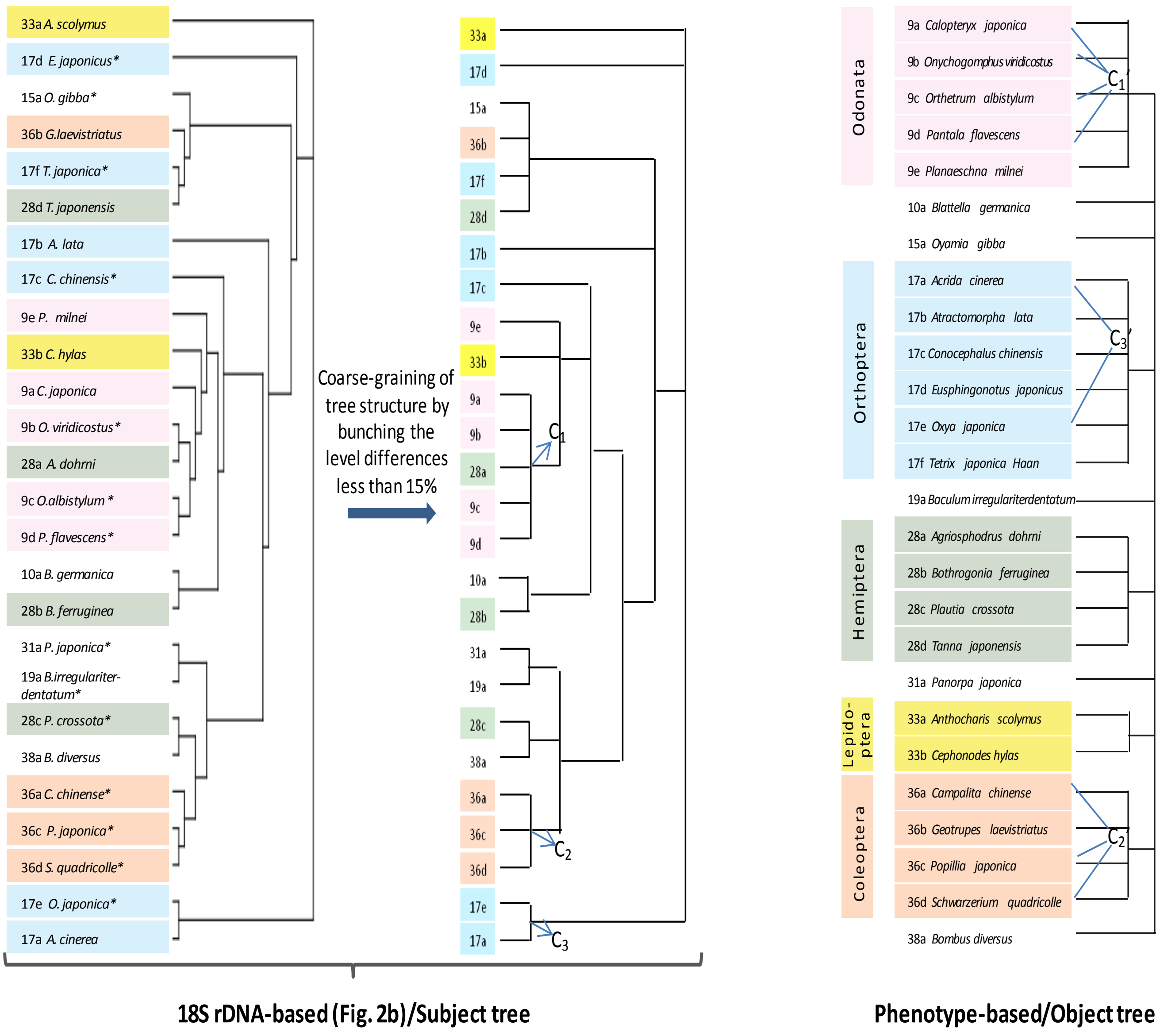

CMS and the number of branches for the pair of phenotype and 18S rDNA-based (Fig. 2c) trees:

For Case π**;**

Tree name	Assigned cluster	Elements	CMS (Eq. adopted)	No. of branches
Phenotype-based tree (Subject)	C_1	9a, 9b, 9c, 9d, 9e	5 (Eq. 1)	=28.5
	C_2	36a, 36b, 36c, 36d	4 (Eq. 1)	
18S rDNA-based tree (Object)	C_1’	9a, 9b, 9c, 9d	5/4=0.8(Eq. 3)	=29.87
	C_2’	36a, 36c, 36d	¾=0.75(Eq. 3)	
**Total**			**10.55**	**58.37**

For Case (-π)**;**

Tree name	Assigned cluster	Elements	CMS (Eq. adopted)	No. of branches
18S rDNA-based tree (Subject)	C_1	9a, 9b, 28a, 9c, 9d	5 (Eq. 1)	=29.87
	C_2	36a, 36c, 36d	3 (Eq. 1)	
	C_3	17e, 17a	2 (Eq. 1)	
Phenotype-based tree (Object)	C_1’	9a, 9b, 9c, 9d	4/5=0.8 (Eq. 3)	=28.5
	C_2’	36a, 36c, 36d	¾= 0.75 (Eq. 3)	
	C_3’	17e, 17a	<0.05	
**Total**			**11.55**	**58.37**

Congruence value (V_c’):

$$V_c' = (10.55 + 11.55)/(2 \times 58.37) = 22.1/116.7 = 0.19$$

4.6 Congruence value (V_c') between phenotype and GP-based (Fig. 3) trees after the coarse-graining process:

Case π of Phenotype-based tree as subject one;

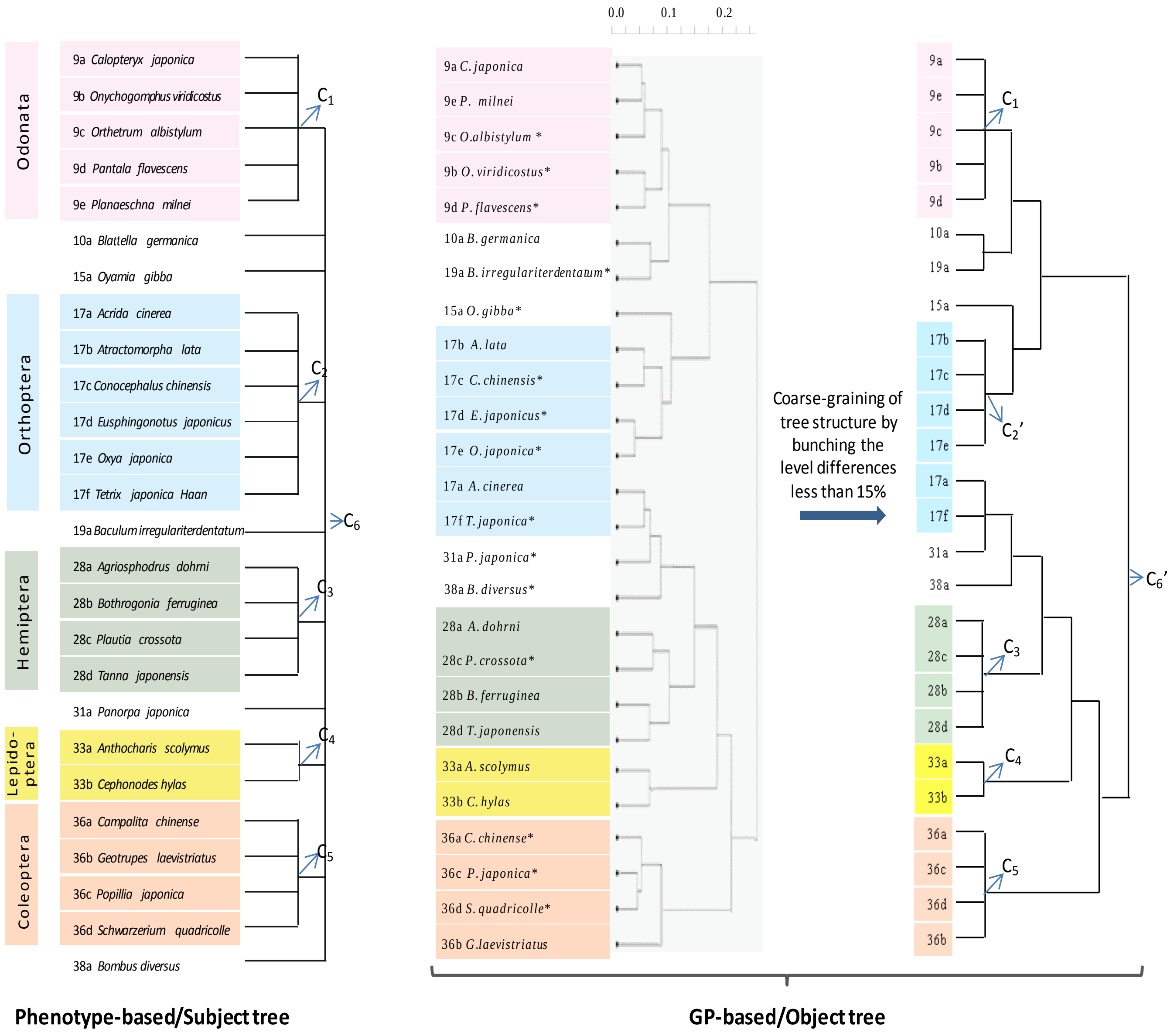

Case (-π) of GP-based as subject one;

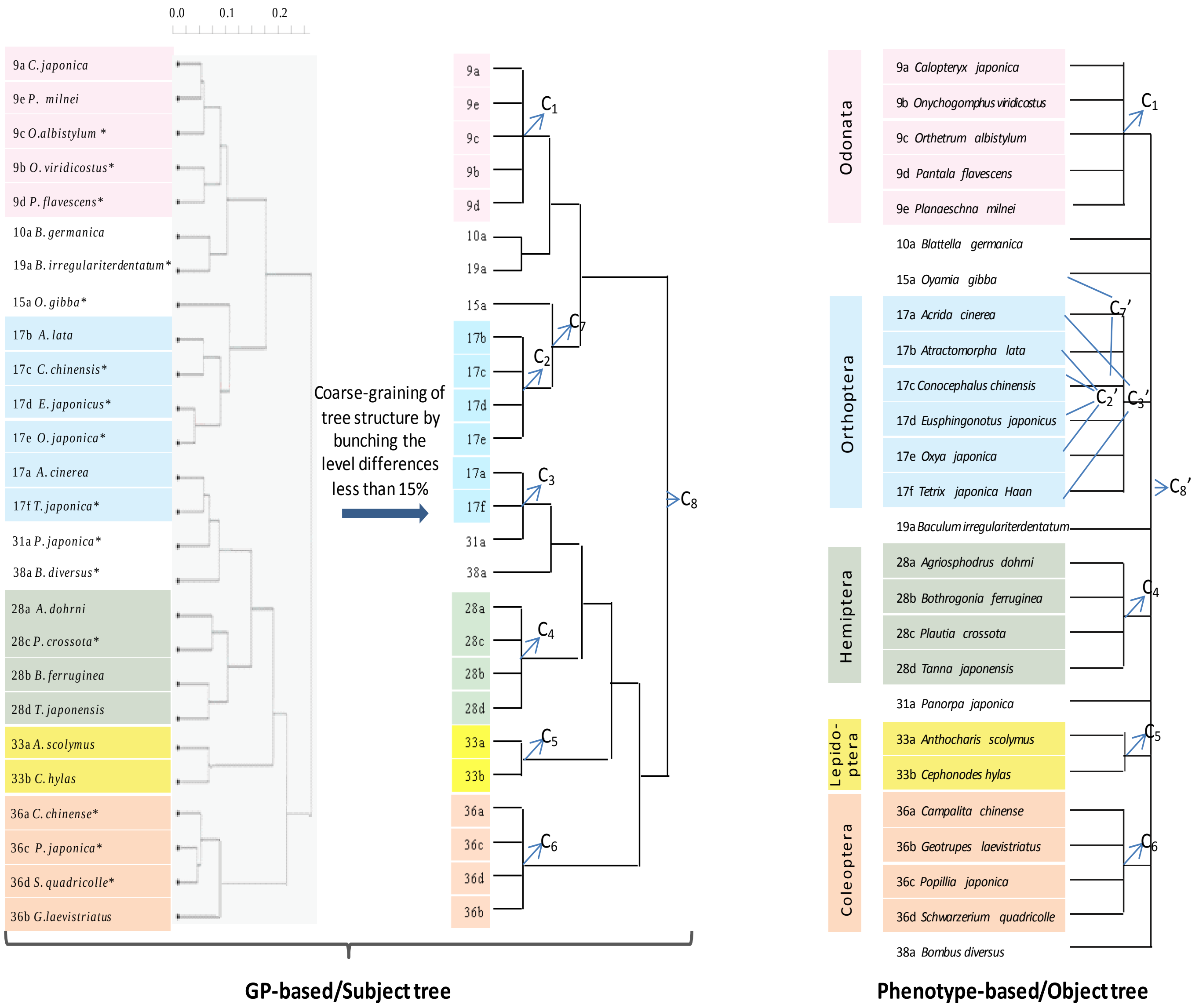

CMS and the number of branches for the pair of phenotype and GP-based (Fig. 3) trees:

For Case π;

Tree name	Assigned cluster	Elements	CMS (Eq. adopted)	No. of branches
Phenotype-based tree (Subject)	C_1	9a, 9b, 9c, 9d, 9e	5 (Eq. 1)	=28.5
	C_2	17a,17b,17c,17d,17e,17f	6 (Eq. 1)	
	C_3	28a, 28b, 28c, 28d	4 (Eq. 1)	
	C_4	33a, 33b	2 (Eq. 1)	
	C_5	36a, 36b, 36c, 36d	4 (Eq. 1)	
	C_6	C_1,10a,15a, C_2, 19a, C_3, 31a, C_4, C_5, 38a	1+.5+.5+1+.5+1+.5+1+1+ .5=7.5	
GP-based tree (Object)	C_1	9a, 9b, 9c, 9d, 9e	5 (Eq. 1)	=36.12
	C_2'	17b, 17c, 17d, 17e	4/30=0.13(Eq. 3)	
	C_3	28a, 28b, 28c, 28d	4 (Eq. 1)	
	C_4	33a, 33b	2 (Eq. 1)	
	C_5	36a, 36b, 36c, 36d	4 (Eq. 1)	
	C_6'	C_1,10a,19a,15a, C_2', 17a, 17f, 31a, 38a, C_3, C_4, C_5	<.05+<.05………	
**Total**			**43.63**	**64.62**

For Case (-π);

Tree name	Assigned cluster	Elements	CMS (Eq. adopted)	No. of branches
GP-based tree (Subject)	C_1	9a, 9b, 9c, 9d, 9e	5 (Eq. 1)	=36.12
	C_2	17b, 17c, 17d, 17e	4 (Eq. 1)	
	C_3	17a, 17f, 31a	3 (Eq. 1)	
	C_4	28a, 28c, 28b, 28d	4 (Eq. 1)	
	C_5	33a, 33b	2 (Eq. 1)	
	C_6	36a, 36c, 36d, 36b	4 (Eq. 1)	
	C_7	C_2, 15a	1+.5= 1.5 (Eq. 2)	
	C_8	C_1,10a, 19a, C_7, C_2, C_3, 38a, C_4, C_5, C_6	~1.0 (Eq. 2)	
Phenotype-based tree (Object)	C_1	9a, 9b, 9c, 9d, 9e	5 (Eq. 1)	=28.5
	C_2'	17b, 17c, 17d, 17e	4/30=0.13(Eq. 3)	
	C_3'	17a, 17f	<0.05	
	C_4	28a, 28b, 28c, 28d	4 (Eq. 1)	
	C_5	33a, 33b	2 (Eq. 1)	
	C_6	36a, 36b, 36c, 36d	4 (Eq. 1)	
	C_7'	15a, C_2'	1+.5=1.5(Eq. 2)	
	C_8'	C_1,10a, 15a, C_2', C_3', C_7', C_4, C_5, C_6, 38a,	1+.5+.5+1+1+1+1+1+1+. 5=8.5	
**Total**			**47.13**	**64.62**

Congruence value (V_c'):

$$V_c' = (43.63 + 47.13)/(2 \times 64.62) = 91.8/129 = 0.71$$

Summary findings of the current study:

Trees	V_c	V_c'
Phenotype vs 18S rDNA-based tree (Fig. 2b, 26 samples)	0.06	0.19
Phenotype vs 18S rDNA-based tree (Fig. 2c, 16 samples)	0.26	0.73
Phenotype vs GP-based tree (Fig. 3)	0.24	0.71
