# Supplementary material for: Genome Profiling (GP) Method Based Classification of Insects: Congruence with That of Classical Phenotype-Based One
Source: PLoS One. 2011 Aug 31;6(8):e23963. doi: 10.1371/journal.pone.0023963 (PMC3166070; doi:10.1371/journal.pone.0023963)
Supplement: Table S1 — Taxonomy† of the species dealt in this study. (DOC) [file pone.0023963.s005.doc]

| No. | Species / Conventional name | Family | Order | Calss | Phylum |
| --- | --- | --- | --- | --- | --- |
| A1 | *Typha orientalis* / Bulrush sp. | Typhaceae | Typhales | Mono^*^ | Anth* |
| A2 | *Arundinaria argenteostriata* / Bamboo sp. | Poaceae | Cyperales | Mono^*^ | Anth* |
| A3 | *Tricyrtis hirta* / Lily sp. | Liliaceae | Liliales | Mono^*^ | Anth* |
| A4 | *Cosmos bipinnatus /* Cosmos sp. | Asteraceae | Asterales | Dico^*^ | Anth* |
| A5 | *Taraxacum officinale /* Dandelion sp. | Asteraceae | Asterales | Dico^*^ | Anth* |
| A6 | *Callicarpa dichotoma* / Beauty-berry sp. | Verbenaceae | Lamiales | Dico^*^ | Anth* |
| A7 | *Gardenia jasminoides* / Gardenia sp. | Rubiaceae | Rubiales | Dico^*^ | Anth* |
| A8 | *Papaver nudicaule* / Poppy sp. | Papaveraceae | Papaverales | Dico^*^ | Anth* |
| A9 | *Viola xwittrockiana* / Pansy sp. | Violaceae | Violales | Dico^*^ | Anth* |
| A10 | *Camellia sasanqua* / Camellia sp. | Theaceae | Theales | Dico^*^ | Anth* |
| A11 | *Davidia involucrata* / Dove tree sp. | Davidiaceae | Cornales | Dico^*^ | Anth* |
| A12 | *Hydrangea macrophylla* / Hydrangea sp. | Hydrangeaceae | Rosales | Dico^*^ | Anth* |
| B1 | *Chilocorus rubidus* / Beetle sp. 1 | Coccinellidae | Coleoptera | Inse^*^ | Arth^*^ |
| B2 | *Oxycetonia jucunda* / Beetle sp. 2 | Scarabaeidae | Coleoptera | Inse^*^ | Arth^*^ |
| B3 | *Bombylius major* / Horse fly sp. | Bombyliidae | Diptera | Inse^*^ | Arth^*^ |
| B4 | *Camponotus japonicus* / Ant sp. 1 | Formicidae | Hymenoptera | Inse^*^ | Arth^*^ |
| B5 | *Formica japonica* / Ant sp. 2 | Formicidae | Hymenoptera | Inse^*^ | Arth^*^ |
| B6 | *Apis mellifera* / Bee sp. | Apidae | Hymenoptera | Inse^*^ | Arth^*^ |
| B7 | *Limenitis camilla* / Butterfly sp. 1 | Nymphalidae | Lepidoptera | Inse^*^ | Arth^*^ |
| B8 | *Anthocharis scolymus* / Butterfly sp. 2 | Pieridae | Lepidoptera | Inse^*^ | Arth^*^ |
| B9 | *Pieris rapae crucivora* / Butterfly sp. 3 | Pieridae | Lepidoptera | Inse^*^ | Arth^*^ |
| B10 | *Eurema laeta* / Butterfly sp. 4 | Pieridae | Lepidoptera | Inse^*^ | Arth^*^ |
| B11 | *Gonolabis marginalis* / Earwig sp. | Anisolabididae | Dermaptera | Inse^*^ | Arth^*^ |
| B12 | *Bothrogonia ferruginea* / Stinkbug sp. | Cicadellidae | Hemiptera | Inse^*^ | Arth^*^ |
| B13 | *Blattella germanica* / Cockroach sp. | Blattellidae | Blattaria | Inse^*^ | Arth^*^ |
| B14 | *Reticulitermes speratus* / Termite sp. | Rhinotermitidae | Isoptera | Inse^*^ | Arth^*^ |
| C1 | *Oncorhynchus masou* / Salmon sp. 1 | Salmonidae | Salmoniformes | Acti^*^ | Chor^*^ |
| C2 | *Oncorhynchus tshawytscha* / Salmon sp. 2 | Salmonidae | Salmoniformes | Acti^*^ | Chor^*^ |
| C3 | *Oncorhynchus mykiss* / Rainbow trout | Salmonidae | Salmoniformes | Acti^*^ | Chor^*^ |
| C4 | *Salmo trutta* / Brown trout | Salmonidae | Salmoniformes | Acti^*^ | Chor^*^ |
| C5 | *Salvelinus malma malma*/ Dolly Varden | Salmonidae | Salmoniformes | Acti^*^ | Chor^*^ |
| C6 | *Salvelinus leucomaenis* / Whitespotted char | Salmonidae | Salmoniformes | Acti^*^ | Chor^*^ |
| C7 | *Hucho perryi* / Japanese huchen | Salmonidae | Salmoniformes | Acti^*^ | Chor^*^ |
| C8 | *Osmerus eperlanus mordax* / Rainbow smelt | Osmeridae | Salmoniformes | Acti^*^ | Chor^*^ |
| C9 | *Cyprinus carpio* / Carp sp. 1 | Cyprinidae | Cypriniformes | Acti^*^ | Chor^*^ |
| C10 | *Phoxinus percnurus* / Carp sp. 2 | Cyprinidae | Cypriniformes | Acti^*^ | Chor^*^ |
| C11 | *Misgurnus anguillicaudatus* / loach sp. 1 | Cobitidae | Cypriniformes | Acti^*^ | Chor^*^ |
| C12 | *Barbatula barbatula* / loach sp. 2 | Balitoridae | Cypriniformes | Acti^*^ | Chor^*^ |
| C13 | *Silurus asotus* / Amur cat fish sp. | Siluridae | Siluriformes | Acti^*^ | Chor^*^ |
| C14 | *Cottus nozawae* / Bullhead sp. | Cottidae | Scorpaeniformes | Acti^*^ | Chor^*^ |

† This table is built based on NCBI’s Taxonomy (http://www.ncbi.nlm.nih.gov/entrez/query.fcgi?CMD=search&DB=taxonomy) and Iwanami Biology Encyclopedia, 4th edition [30]. * Mono: Monocotyledonopsida, Dico: Dicotyledonopsida, Anth: Anthophyta, Inse :Insecta, Acti : Actinopterygii, Chor : Chordata.
