# Supplementary material for: Genome Profiling (GP) Method Based Classification of Insects: Congruence with That of Classical Phenotype-Based One
Source: PLoS One. 2011 Aug 31;6(8):e23963. doi: 10.1371/journal.pone.0023963 (PMC3166070; doi:10.1371/journal.pone.0023963)
Supplement: Table S2 — Tentative comparison in terms of cost, labor and other consumables between 18S rDNA sequencing and GP experiments. (DOC) [file pone.0023963.s006.doc]

|  | 18S rDNA sequencing | GP experiment |
| --- | --- | --- |
| Experimental cost per organism | $30 | $3 |
| Time required for a single experiment | 22 hours | 4 hours |
| Reagents used | PCR reagents, cloning vector, plasmid culture medium, plasmid purification kit, cycle sequencing kit | PCR reagents, acrylamide gel and buffers |

*The base of cost estimation is shown in Supplementary table 3.
