# Supplementary material for: Genome Profiling (GP) Method Based Classification of Insects: Congruence with That of Classical Phenotype-Based One
Source: PLoS One. 2011 Aug 31;6(8):e23963. doi: 10.1371/journal.pone.0023963 (PMC3166070; doi:10.1371/journal.pone.0023963)
Supplement: Table S3 — The basic data for tentative estimation of experimental cost in Yen (Japan, 2009). (DOC) [file pone.0023963.s007.doc]

| **Name of reagents** | **Providers** | | **Kit size** | **Price** | | | **Cost for single experiment** | | |
| --- | --- | --- | --- | --- | --- | --- | --- | --- | --- |
|  | |  |  |  | | | 18S rDNA sequencing | GP experiments | |
| PCR reagents | | Takara, Japan | 1 kit (250 unit) | | 27,000 | | 108 | 108 | |
| Primers | | Tsukuba Oligo Service, Japan | 450 unit | 30,000 | | | 66 | 66 | |
| P^GEM-T^ easy vector with DH5α | | Promega, Japan | 1 kit (20 unit) | 30,000 | | | 1500 |  | |
| Plasmid purification kit | | Promega, Japan | 1 kit (250 unit) | 44,000 | | | 176 |  | |
| Sequencing | | Bio Matrix Research, Japan | 96 tests | 72,000 | | | 750 |  | |
| Others (Tips, acryl amide, urea, etc.) | | - | - | - | | | 50 | 30 | |
|  | |  |  |  | | **Total: 2650 yen*** | | | **204 yen** |

*Currency conversion on March 23, 2010: 90 yen (Japan) = 1 USD, 2650 yen = 29.4 USD
