# Supplementary material for: Genome Profiling (GP) Method Based Classification of Insects: Congruence with That of Classical Phenotype-Based One
Source: PLoS One. 2011 Aug 31;6(8):e23963. doi: 10.1371/journal.pone.0023963 (PMC3166070; doi:10.1371/journal.pone.0023963)
Supplement: Table S4 — Genome sources. (DOC) [file pone.0023963.s008.doc]

| Taxon No. | Name of Species | Obtained from | Accession number |
| --- | --- | --- | --- |
| 9a | *Calopteryx　japonica* | NCBI | Y 12893 |
| 9b | *Onychogomphus viridicostus* | Our lab | HQ630633 |
| 9c | *Orthetrum　albistylum* | Our lab | HQ630634 |
| 9d | *Pantala　flavescens* | Our lab | HQ630635 |
| 9e | *Planaeschna　milnei* | Our lab | HQ630636 |
| 10a | *Blattella　germanica* | NCBI | EF 363236 |
| 15a | *Oyamia　gibba* | Our lab | HQ630637 |
| 17a | *Blattella　germanica* | NCBI | AY 379759 |
| 17b | *Atractomorpha lata* | NCBI | AY 626906 |
| 17c | *Conocephalus chinensis* | Our lab | HQ630638 |
| 17d | *Eusphingonotus　japonicus* | Our lab | HQ630639 |
| 17e | *Oxya　japonica* | Our lab | HQ630640 |
| 17f | *Tetrix　japonica　Haan* | Our lab | HQ630641 |
| 19a | *Baculum irregulariterdentatum* | Our lab | HQ630642 |
| 28a | *Agriosphodrus　dohrni* | Our lab | HQ630643 |
| 28b | *Bothrogonia　ferruginea* | Our lab | HQ630644 |
| 28c | *Plautia　crossota* | Our lab | HQ630645 |
| 28d | *Tanna　japonensis* | Our lab | HQ630646 |
| 31a | *Panorpa　japonica* | Our lab | HQ630647 |
| 33a | *Anthocharis　scolymus* | Our lab | HQ630648 |
| 33b | *Cephonodes hylas* | Our lab | HQ630649 |
| 36a | *Campalita　chinense* | Our lab | HQ630650 |
| 36b | *Geotrupes　laevistriatus* | Our lab | HQ630651 |
| 36c | *Popillia　japonica* | Our lab | HQ630652 |
| 36d | *Schwarzerium　quadricolle* | Our lab | HQ630653 |
| 38a | *Bombus diversus* | Our lab | HQ630654 |
